# The recombinant l-lysine α-oxidase from the fungus *Trichoderma harzianum* promotes apoptosis and necrosis of leukemia CD34 + hematopoietic cells

**DOI:** 10.1186/s12934-024-02315-2

**Published:** 2024-02-14

**Authors:** Mariana do Nascimento Costa, Thiago Aparecido Silva, Dimitrius Santiago Passos Simões Fróes Guimarães, Rafael Ricci-Azevedo, Felipe Roberti Teixeira, Leonardo Reis Silveira, Marcelo Damário Gomes, Vítor Marcel Faça, Eduardo Brandt de Oliveira, Rodrigo T. Calado, Roberto N. Silva

**Affiliations:** 1 Department of Biochemistry and Immunology, Ribeirão Preto Medical School, University of São Paulo, Ribeirão Preto, SP Brazil; 2 Department of Cell Biology and Molecular and Pathogenic Bioagents, Ribeirão Preto Medical School, University of São Paulo, Ribeirão Preto, SP Brazil; 3 Department of Clinical Analysis, School of Pharmaceutical Sciences in Araraquara, Sao Paulo State University, Araraquara, SP Brazil; 4 Obesity and Comorbidities Research Center, Institute of Biology, University of Campinas, Campinas, SP Brazil; 5 Department of Genetics and Evolution, Center of Biological and Health Sciences, Federal University of São Carlos, São Carlos, SP Brazil; 6 Department of Medical Imaging, Hematology, and Oncology, Ribeirão Preto Medical School, University of São Paulo, Ribeirão Preto, SP Brazil

**Keywords:** Leukemia, l-lysine α-oxidase, *Trichoderma harzianum*, Cancer treatment

## Abstract

**Background:**

In hematologic cancers, including leukemia, cells depend on amino acids for rapid growth. Anti-metabolites that prevent their synthesis or promote their degradation are considered potential cancer treatment agents. Amino acid deprivation triggers proliferation inhibition, autophagy, and programmed cell death. l-lysine, an essential amino acid, is required for tumor growth and has been investigated for its potential as a target for cancer treatment. l-lysine α-oxidase, a flavoenzyme that degrades l-lysine, has been studied for its ability to induce apoptosis and prevent cancer cell proliferation. In this study, we describe the use of l-lysine α-oxidase (LO) from the filamentous fungus *Trichoderma harzianum* for cancer treatment.

**Results:**

The study identified and characterized a novel LO from *T. harzianum* and demonstrated that the recombinant protein (rLO) has potent and selective cytotoxic effects on leukemic cells by triggering the apoptotic cascade through mitochondrial dysfunction.

**Conclusions:**

The results support future translational studies using the recombinant LO as a potential drug for the treatment of leukemia.

**Supplementary Information:**

The online version contains supplementary material available at 10.1186/s12934-024-02315-2.

## Background

The survival of any organism is dependent on regulated cell proliferation, differentiation, and death. These processes are affected by intracellular and environmental factors contributing to cellular homeostasis, which, once unbalanced, changes the regulatory mechanisms involved in cell growth and survival that can result in the alteration of proliferation rate and/or cellular differentiation generating a neoplasm [1].

Hanahan and Weinberg hypothesized the main eight changes in cell physiology that collectively promote malignant cell growth, including dysregulation of cellular energetics [2]. This condition can be demonstrated by the Warburg effect, or anaerobic glycolysis, which is characterized by the increased glycolytic metabolism of tumor cells even in the microenvironment with high energy demand and low oxygen pressure. Anaerobic glycolysis promotes lower ATP production yield than oxidative phosphorylation. However, the glycolysis intermediates still maintain a microenvironment able to synthesize nucleotides, amino acids, and macromolecules [3, 4]. On the other hand, aerobic glycolysis allows an increase in the amino acid catabolism. Also, it induces a higher expression of glucose and amino acid transporters in the tumor cell surface to uptake more nutrients from the bloodstream [5]. These tumor adaptations have created a potential target for cancer treatment based on the metabolic differences between cancerous and healthy cells. Reducing the availability of specific amino acids, such as l-lysine, can promote cancer cell cycle arrest, preventing disease progression, while preserving normal cells, which have a lower requirement of this amino acid as a precursor in the synthesis of new compounds [6]. Therefore, the regulatory function of amino acids over the proliferation of tumor cells and the amino acid deprivation triggers the inhibition of cellular proliferation and induces autophagy [7, 8]. When these mechanisms fail to supply amino acids, cells undergo programmed cell death [9].

The so-called ‘anti-metabolites’ are drugs capable of preventing the synthesis or promoting the depletion of essential molecules for cell proliferation, such as nitrogenous bases, nucleotides, and amino acids [6]. Hematological neoplasm requires a high amount of non-essential amino acids, such as asparagine, and essential ones, like l-lysine, arginine, and methionine, from external sources [10]. Cancer therapies targeting amino acid deprivation renewed the interest in microbial enzymes such as l-asparaginase, arginine deiminase, methionase, and l-lysine α-oxidase. l-asparaginase is a member of the amidohydrolase family and it is the first enzyme approved for cancer therapy. L-asparaginases has been produced in *E. coli* and *Erwinia chrysanthemi* in its native and recombinant form for treating acute lymphocytic leukemia [11].

Both l-lysine and l-arginine are required for rapid tumor growth, as they are important constituents of the proteins that organize chromatin in the cell nucleus. The role of l-lysine in numerous cellular processes contributes to its placement as a strong target for cancer treatment [12]. As an essential amino acid, l-lysine cannot be de novo synthesized for metabolic needs and must be absorbed from the diet [13]. Studies on the effect of l-lysine on cancer proliferation go back to the beginning of the twentieth century when in 1915, Kocher observed that, when compared with normal tissues, the concentration of l-lysine, l-arginine, and l-histidine was more than twofold higher in cancer [14, 15]. The removal of l-lysine from the culture medium has been shown to reduce the proliferation of leukemic cells, indicating that an abundance of l-lysine is required for tumor cell growth. Accordingly, a study showed that depleting 80% of l-lysine from the blood was sufficient to promote a decrease in leukocyte concentration and a reduction in the proliferative capacity of these cells [16].

l-lysine α-oxidase (LO EC 1.4.3.14) or l-lysyl α-oxidase is a flavoenzyme of the l-amino acid oxidase (LAAO) family, which catalyzes the oxidative deamination of l-amino acids producing α-keto acid, hydrogen peroxide, and ammonia [15]. LAAO enzymes are widely distributed in nature and are usually involved in defense mechanisms against parasites and other threats. The pharmacological application of LAAOs’ ability to induce apoptosis and hinder proliferation has been investigated in several studies. Noticeably, these outcomes are caused not only by the amino acid removal but also due to the secondary effect of hydrogen peroxide produced during the enzymatic reaction that induces oxidative stress in these cells [14].

Although there is a considerable volume of scientific literature investigating and characterizing these enzymes capable of breaking down amino acids, due to their promising application in the biomedical area, the medicinal of LAAOs is still impractical, given the high cost of isolating these proteins from their natural sources [17]. On the other hand, using protein engineering tools makes amino acid-depleting enzymes an appealing treatment. In this context, l-lysine α-oxidase obtained from *Trichoderma harzianum* exhibits a high cytotoxic and antiproliferative effect in several tumor types like prostate cancer, ovarian cancer, breast cancer, and myeloid leukemia [18].

*Trichoderma harzianum* is a filamentous fungus used as a biological agent against phytopathogens such as *Botrytis*, *Rhizoctonia*, and *Fusarium* which cause significant damage to several crops [19]. The mycoparasitic activity of some species of *Trichoderma* involves not only physical contact but also the synthesis of lytic enzymes such as l-lysine oxidase and other toxic components [20].

This report identified and characterized a novel LO from *T. harzianum*. The enzyme coding sequence was cloned, and the recombinant protein produced in *E. coli* was used to treat cells from healthy and leukemic donors. We show that rLO has a potent and selective cytotoxic effect on leukemic cells. Specifically, rLO treatment can trigger the apoptotic cascade by mitochondrial dysfunction.

## Results

### Cloning and heterologous production of rLO

*Trichoderma* l-amino acid oxidases (LAAOs) have been studied in a variety of contexts. Specifically, culture fluids or l-lysine α-oxidase activity-enriched fractions were used as antibacterial, antifungal, and antitumoral agents [21]. Thus, we sought to identify and characterize *T. harzianum* ALL42 l-Lysine α-oxidase. The fungus was cultured by fermentation in a semi-solid state in the presence of wheat bran, and native LO was partially purified by ion exchange chromatography followed by molecular exclusion with a yield of 38% and final LO specific activity of 26.11 U/mg (Additional file 1: Fig. S1, Table S1). The residual sequence obtained by mass spectrometry of the partially purified enzyme pointed to the gene aox1, a *putative l-amino acid oxidase from* *Trichoderma harzianum*, with 97% identity after BLASTn search (Additional file 1: Table S2).

**Fig. 1:**
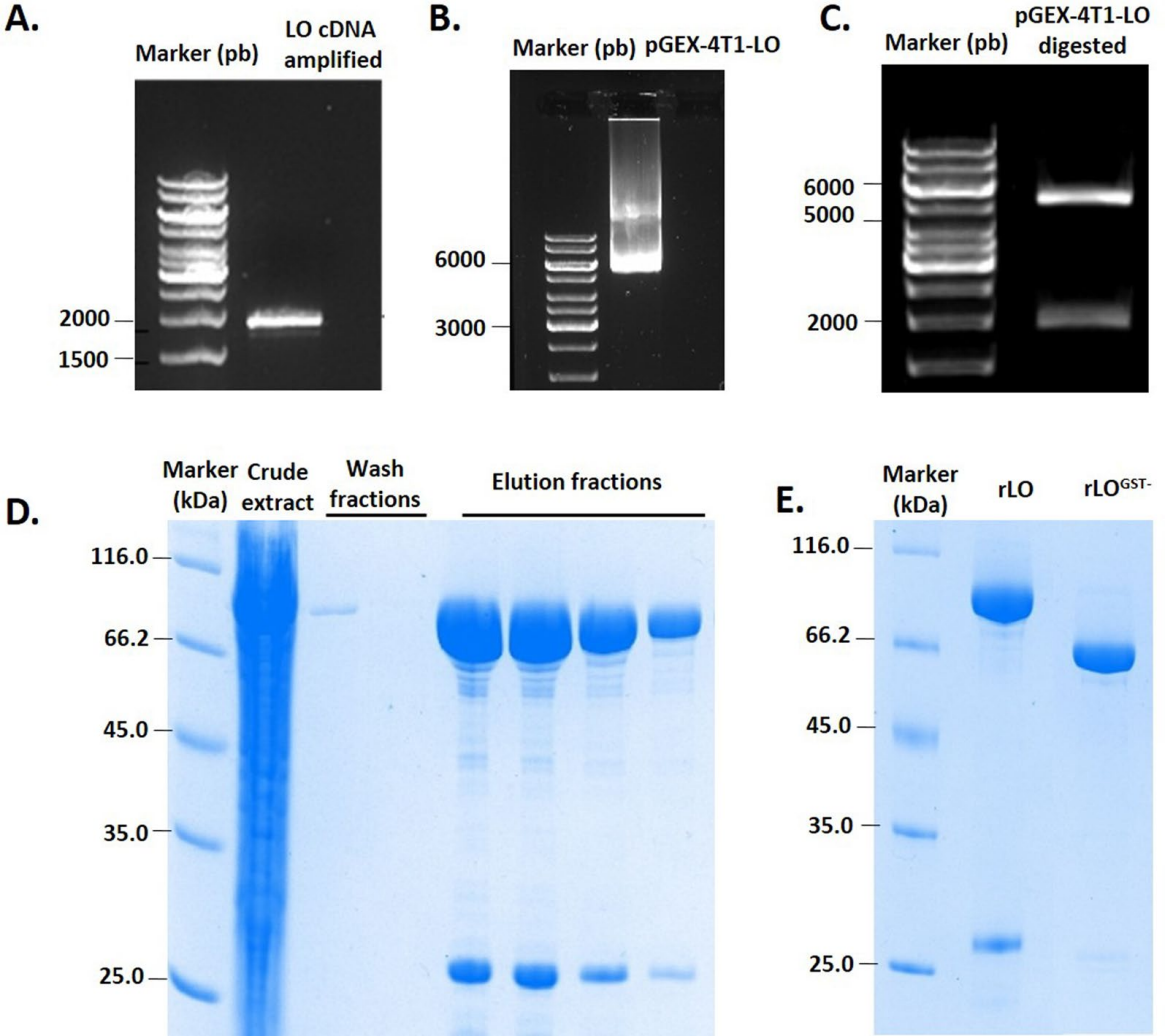
Cloning and heterologous production of rLO. **A** Gel 1% agarose of PCR amplification of LO gene. Line 1: DNA marker; Line 2: Band of LO gene amplified from the cDNA of *T. harzianum*. **B** Gel 1% agarose of the vector cloned pGEX-4T1-LO. **C** Gel 1% agarose of the fragments of plasmid pGEX-4T1 and LO gene after plasmid cleavage with the restriction enzymes *BamHI* and *EcoRI* confirming the fragments with the expected sizes. **D** SDS-PAGE of rLO purification. Line 1: Protein marker; Line 2: Crude extract; Line 2 to 3: Wash samples; Line 4 to 15: Elution samples. The band between 66.2 and 116 kDa is rLO. The band below is GST. **E** Gel SDS-PAGE of rLO (Line 2) and rLO^GST−^ (Line 3) after treatment of rLO with Thrombin Protease for GST removal. Line 1: Protein Marker. Molecular mass standards: β-galactosidase, 116 kDa; bovine serum albumin, 66.2 kDa; ovalbumin, 45 kDa; lactate dehydrogenase, 35 kDa; REase Bsp98I, 25 kDa; β-lactoglobulin, 18.4 kDa; lysozyme, 14.4 kDa

The LO gene from *T. harzianum* was amplified by PCR from cDNA and then cloned into the expression vectors pET28a (His-tag) and pGEX-41T (GST-tag). The correct frame and sequence were confirmed by sequencing. Initial results showed no enzymatic activity in the crude extract of *E. coli* transformed with the pET28a-rLO construct. Also, we observed an accumulation of proteins in the insoluble fraction (data not shown). On the other hand, the recombinant l-lysine α-oxidase (rLO) from pGEX-41T was expressed in the *E. coli* BL21 strain and remained in the soluble fraction with high enzymatic activity. SDS-PAGE gel of affinity purified rLO (Fig. 1) shows the bands before (Band A—rLO) and after GST tag removal (Band B—rLO^GST−^), migrating as individual bands of 86 kDa and 60 kDa, respectively. Kinetic characterization data of the recombinant enzyme (rLO) is presented in Tables S3, S4 and Figure S4.

### Trichoderma harzianum rLO cytotoxicity in Jurkat cells derived from acute T-cell leukemia

**Fig. 2:**
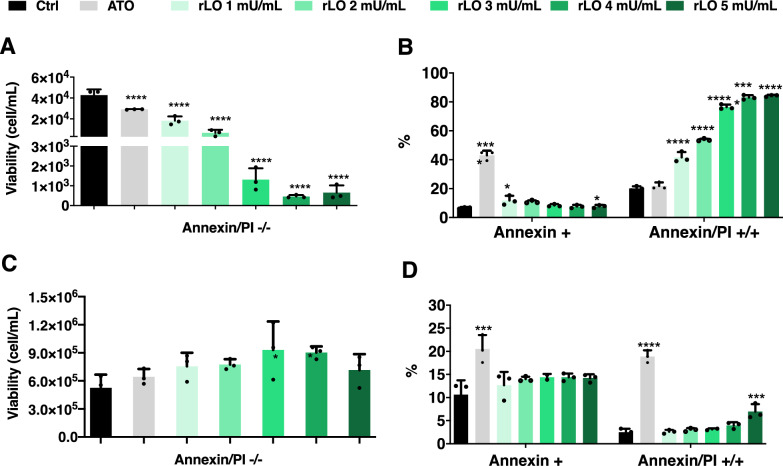
Evaluation of cytotoxicity of rLO in Jurkat and PBMC cells. Jurkat cells (**A**, **B**) and PBMC (**C**, **D**) were treated with doses of 1 to 5 mU/mL of rLO, and after 24 h of incubation, the cells were stained with annexin V and/or PI following the flow cytometry analysis. Arsenic trioxide (ATO) was considered a positive control, and the negative control was growth medium only. **A** and **C** The concentration of viable cells was determined by the number of negative cells for unstained cells as measured by flow cytometry. **B** and **D** Percentage of cells stained for early apoptosis (Annexin V +) and late apoptosis (Annexin V/PI + / +) after the treatment with different doses of rLO

l-lysine depletion can inhibit aggressive proliferation that usually occurs in leukemia cells, therefore, we investigated the effects of rLO (1 mU/mL to 5 mU/mL) in the induction of apoptosis of the acute leukemia T-cell line Jurkat. Moreover, peripheral blood mononuclear cells (PBMC) were used to assess the rLO cytotoxic activity over immune cells obtained from healthy donors (Fig. 2). Jurkat cells were treated with increasing concentrations of rLO, and the early-stage apoptosis and late-stage apoptosis were determined by Annexin V-FITC and/or PI staining after 24 h of incubation using flow cytometry. Jurkat cells growing in medium alone were considered as a negative control, and cells treated with arsenic trioxide (ATO), a known antileukemic drug, as a positive control. ATO reduces cell viability in a dose dose-dependent manner and the concentration used allows the detection of both necrosis and apoptosis without excessive cell death in both Jurkat and PBM cells.

rLO significantly reduced the concentration of viable cells in a dose-dependent manner, and the lowest concentration of rLO provided a 58% decrease in viability compared to untreated cells (Fig. 2A). Moreover, the highest dose of rLO reached a 98% reduction of viable cells compared to control cells (Fig. 2A). The percentage of annexin V-positive cells was not significantly increased in 2, 3, and 4 mU/mL rLO after 24 h of incubation, compared to untreated cells. A statistical difference was observed in the lowest (1 mU/mL) and highest (5 mU/mL) dose, but the magnitude is not relevant, and it is likely due to reminiscent cells in apoptosis that did not go through necrosis (Fig. 2B). The percentage of annexin V/PI double-positive cells was markedly higher in response to all concentrations of rLO (Fig. 2B), which characterizes a predominance of a late stage of cell death within 24 h.

To validate that the rLO cytotoxicity occurred in Jurkat cells and not in human cells from healthy donors, PBMCs were incubated with different concentrations of rLO.

Except for the highest dose (5 mU/mL) of rLO, which induced a slight increase in annexin V/PI double-positive cells, no significant alterations in the concentration of viable cells (Fig. 2C) or annexin V/PI (Fig. 2D) were observed when compared to PBMC incubated with growth medium only.

To evaluate the influence of GST on the rLO cytotoxicity in Jurkat cells, the GST tag was removed by enzymatic cleavage at the thrombin site between the GST and rLO, and the untagged rLO was named rLO^GST−^. The concentration of viable Jurkat cells decreased after the treatment with the tagged enzyme (rLO) but not by rLO^GST−^, whereas adding the GST tag alone did not show cytotoxic effects in Jurkat cells even after 24 h (Figure S5D). rLO^GST−^ induced an increase in the percentage of Jurkat cells positive for annexin V. However, rLO^GST−^ could not reduce the concentration of viable cells after 24 h treatment (Figure S5A). Enzymatic assays demonstrated that the GST tag improves the catalytic activity of rLO, from 0.6 U/mg (rLO^GST−^) to 2.2 U/mg (rLO^GST+^) whereas the native LO showed 1.488 U/mg of activity. Therefore, these results indicate that somehow GST tag influences the LO structure conformation, maintaining its cytotoxic effect in Jurkat cells. However, more biophysical studies are necessary to elucidate this influence.

### rLO treatment promotes apoptosis in cancer but not healthy cells

**Fig. 3:**
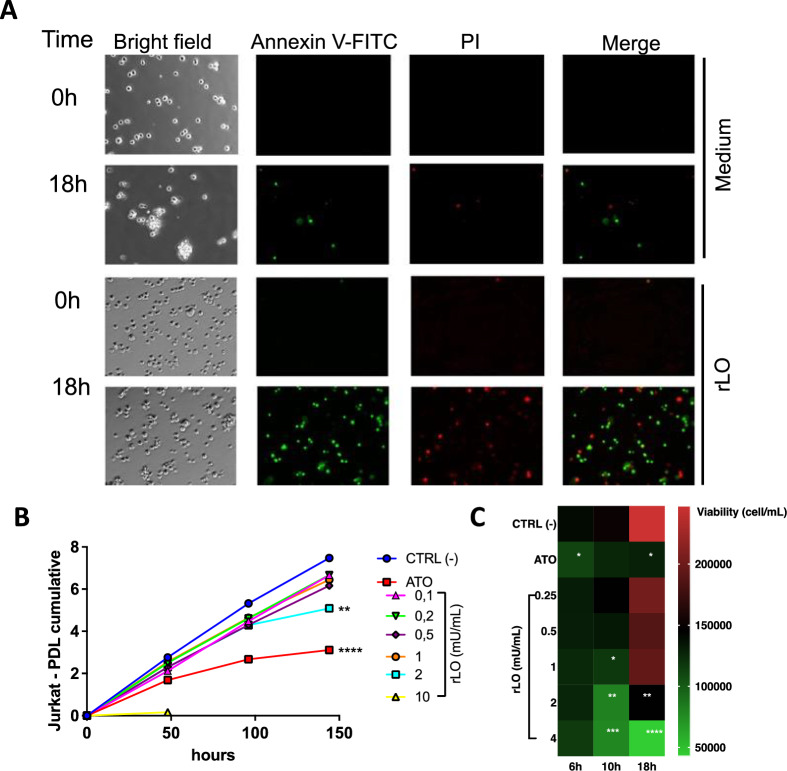
Dose- and time-dependent response of rLO treatment on apoptotic and necrotic events in Jurkat cells. **A** Time-lapse microscopy of rLO-treated Jurkat cells. Photographs were taken every 3 min for 22 h. In the figure, the photos correspond to 0 and 18 h after stimulation with rLO 1 mU/mL. **B** Evaluation of growth inhibition of Jurkat cell by Population Doubling Level (PDL). Jurkat cells were treated with different concentrations of rLO, and growth inhibition was evaluated by counting cells in a Neubauer chamber every 48 h. Negative control: untreated cells. Positive control: cells treated with ATO. **C** Comparative analysis by Heatmap of the viability of Jurkat cells treated with rLO over time. Jurkat cells were treated with doses of 0.25, 0.5, 1, 2, and 4 mU/mL of rLO. After 6, 10, and 18 h, propidium iodide was added to the samples, and viability was determined by analysis of PI incorporation by flow cytometry. CTRL (-): Medium/untreated cells. Positive control: ATO

Since the 24-h treatment with rLO showed high cytotoxicity with late apoptosis events, the viability of Jurkat cells was evaluated using the PI exclusion method in a time-course experiment. Statistical analysis indicated no significant difference in viability between treatments and the negative control within 6 h, except for the positive control, ATO (Fig. 3C). The cytotoxic effect of rLO was observed after 10 and 18 h of treatment at the concentrations of 1, 2, and 4 mU/mL of rLO (Fig. 3C). Also, except for the highest concentration of rLO, cell viability increases after 18 h when compared to the previously evaluated times. We speculate this happens because a subpopulation of cells that had not been affected continued to proliferate, overlapping the rLO’s effect in lower doses.

Time-lapse fluorescence microscopy was performed to show the response of Jurkat cells to rLO over time. The fluorescent markers annexin V-FITC and PI were used as apoptosis and cell death indicators, respectively. The displayed frames (Fig. 3A) show the increase in fluorescence intensity for annexin V (green) and PI (red) starting after 8 h of treatment. The fluorescence intensity of annexin V increases tenfold until 18 h, when there’s an increase in PI fluorescence, indicating that the cells progressed from the initial stages of apoptosis to cell death. Within this same time frame, only isolated events of apoptosis and necrosis were observed in untreated cells. Strikingly, cells treated with rLO also lose their aggregation characteristic, given these cells naturally form small clusters (Fig. 3A, bright field).

Since l-lysine is also necessary for synthesizing nucleotides, l-lysine withdrawal also affects doubling time in fast-growing cells. Therefore, to evaluate whether rLO could also affect the proliferation rate of Jurkat cells, a cumulative population doubling level (PDL) assay was performed for six days. Although lower rLO concentrations could not impact the growth rate, 2 mU/mL rLO treatment reduced the doubling rate by 86% on the sixth day (Fig. 3B).

### *Evaluation of apoptotic and necrotic events in CD34* + *cells treated with rLO—Leukemia patients and healthy donors*

**Fig. 4:**
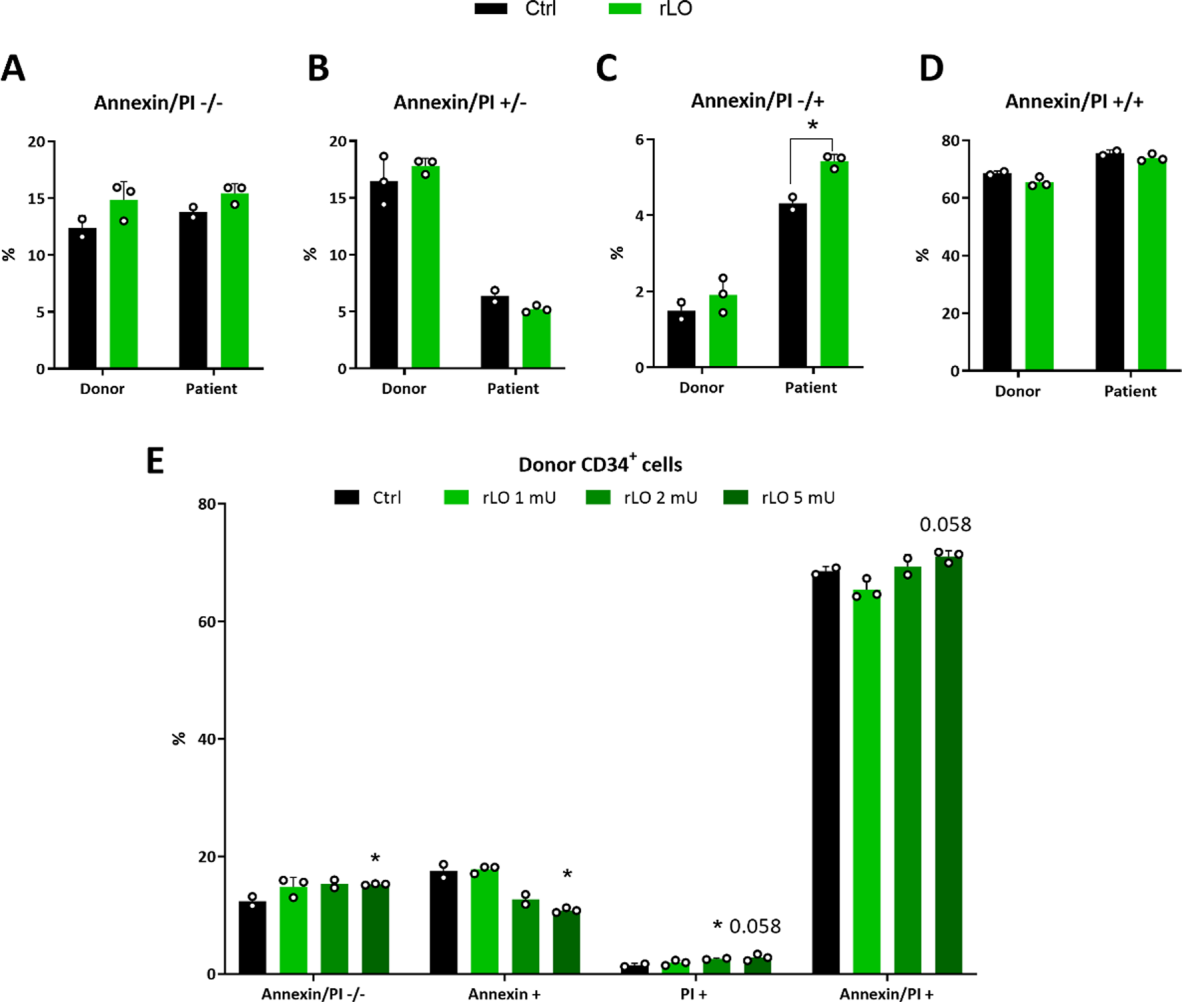
Effects of rLO treatment in the viability of CD34 + cells of leukemic and healthy donors. **A** Percentage of CD34 + viable cells treated with rLO. **B** Percentage of apoptotic CD34 + cells after treatment with rLO. **C** Percentage of necrotic CD34 + cells treated with rLO **D** Percentage of CD34 + cells in late apoptosis after treatment with rLO. **E** Response of healthy CD34 + cells to different concentrations of rLO and percentage of annexin V and PI positive cells. In black: Negative control. Number of patients: 2. Number of healthy donors: 1

Considering the metabolic differences between cell lines and stem cells, regarding their distinct dependency on specific amino acids, we tested whether the rLO cytotoxic effect observed in Jurkat cells could be translated to primary CD34 + hematopoietic stem-progenitor cells from leukemia patients and healthy bone marrow donors. Primary cells were treated with 1 mU/mL of rLO and evaluated for apoptotic and necrotic events. The viability of CD34 + cells of healthy donors and patients was maintained between 12 and 15% after treatment with rLO (Fig. 4A). No statistical difference in annexin V staining was observed between patient and healthy donor cells (Fig. 4B), indicating that rLO could not induce apoptosis in healthy and leukemic CD34 + cells. A significant increase in cell death by necrosis is observed only in the patient’s cells (Fig. 4C), although double labeling showed no significant difference for cells treated with rLO (Fig. 4D). It is important to consider that cell manipulation during the purification of CD34 + cells from donor and patient serum can lead to stress, mainly in healthy cells, explaining the small percentage of apoptotic cells in donor samples.

Different concentrations of rLO (1, 2, and 5 mU/mL) were tested to investigate dose–response effects in CD34 + cells (Fig. 4E). A significant increase in necrotic cells was observed at the highest dose tested. This behavior is different from that observed in mature leukemia cells Jurkat since no apoptotic effect was observed in the latter.

### Determination of the signaling pathways involved in the rLO mechanism of action against Jurkat cells

**Fig. 5:**
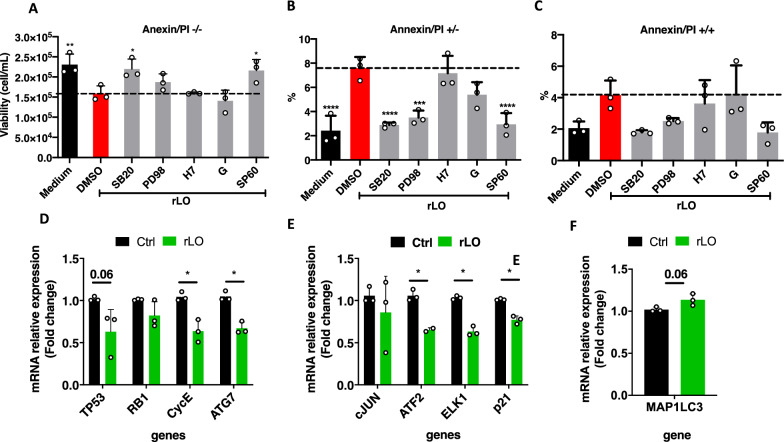
Cell survival and proliferation pathways are reduced in rLO-treated Jurkart cells. **A** Concentration of viable cells. **B** Percentage of apoptotic cells **C** Percentage of late apoptotic cells. **D** Relative expression of genes from JNK pathway **E** Relative expression of genes from p38 pathway. **F** Relative expression of the LC3 gene of the autophagosome formation

Although we know the potential of rLO to promote cell death in Jurkat cells, there is no evidence of its mechanism of action. In this context, the effect of rLO on viability and apoptosis of Jurkat cells was investigated in the presence of the following inhibitors of cellular pathways related to cell proliferation and survival: genistein (PTK inhibitor), PD98059 (ERK inhibitor), SB202190 (p38 MAPK inhibitor), H-7 (PKC inhibitor), SP600125 (JNK inhibitor) or DMSO as a positive control. Cells were incubated with inhibitors for 3.5 h and subsequently treated with 1 mU/mL rLO or medium only. The effect on viability and apoptosis was assessed after 18 h by measuring annexin V/PI staining in a flow cytometer. We assumed that if the effect of rLO has been minimized or abolished in the presence of the inhibitor, it indicates that that pathway is important for the action of the enzyme (Fig. 5).

Among the pharmacological agents, those inhibiting p38 and JNK significantly decreased the effect of rLO in Jurkat cells. However, no changes in the effect of rLO on cell viability were observed following the treatment with PTK, ERK, and PKC inhibitors (Fig. 5A). Furthermore, inhibitors of PTK and PKC did not prevent the apoptosis of Jurkat cells induced by rLO (Fig. 5B). There is no significant difference in the ratios of cell population labeled with both annexin V and PI, indicating that after 18 h of treatment, Jurkat cells have not yet evolved from an initial stage of apoptosis to cell death (Fig. 5C). The expression of downstream genes of the mitogen-activated protein kinases JNK and p38 were analyzed in Jurkat cells treated with 1 mU/mL of rLO. Figure 5D shows a significantly lower expression of CycE and ATG7 [22, 23] involved in the JNK pathway. The genes ATF2, ELK1, and p21 from the p38 pathway [23, 24] also presented a reduced expression when the cells were treated with rLO (Fig. 5E). Interestingly, the expression of the gene that encodes the microtubule-associated protein 1 light chain 3 beta (LC3B), which is a key component of the autophagic membrane, remained unchanged when compared to the control (Fig. 5F). Collectively, these results demonstrate that the induction of necrosis and the reduction of cell viability promoted by rLO is dependent on the MAPK pathway, specifically on p38 and JNK.

### Analysis of respiratory parameters in Jurkat cells treated with rLO

**Fig. 6:**
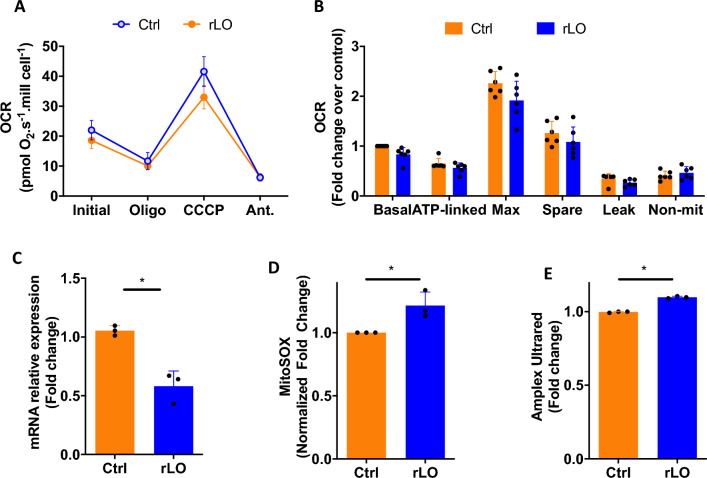
rLO treatment causes acute mitochondrial dysfunction and oxidative stress. **A** Oxygen consumption rates in untreated (Ctrl) and rLO-treated Jurkat cells. **B** Respiratory parameters of oxygen consumption assays. **C** Gene expression of PGC-1α measured by RT-qPCR. **D** Mitochondrial superoxide production was measured with a MitoSOX fluorescent probe. **E** Peroxide production measured by Amplex UltraRed fluorescence intensity

Considering that the impairment of mitochondrial activity is one of the hallmark events that trigger apoptosis, in which the disruption of the intermembrane potential leads to the nucleation of the apoptosome [25], we evaluated the mitochondrial function in Jurkat following rLO treatment. Oxygen consumption assays in cells treated with rLO for 18 h demonstrated a 25% reduction in the maximal respiratory rate and spare capacity compared to controls (Fig. 6A, B). Additionally, there is a strong tendency for a reduction in the basal respiratory rate, indicating a decrease in mitochondrial density. These observations are consistent with the nearly twofold decrease in the expression of the peroxisome proliferator-activated receptor gamma coactivator 1-alpha (PGC-1α) (Fig. 6C), the core regulator of mitochondrial biogenesis and oxidative capacity [26, 27]. Defects in the mitochondrial electron transfer chain can increase the production of reactive oxygen species (ROS), often higher in cancer, rendering these cells more susceptible to the harmful effects of a further increase in ROS levels [28, 29]. Using specific probes for peroxide and mitochondrial superoxide (Fig. 6D, E), we observed a significant increase of approximately 15% in the content of both reactive oxygen species in rLO-treated cells, which is compatible with the impairment in electron transfer chain function. These results indicate that the cytotoxicity of rLO is supported by a dysfunction in mitochondrial efficiency and density or worsened by a dysfunction in metabolic activity and a reduction of mitochondrial density, leading to increased ROS levels and cell death.

## Discussion

Since the 1950s, it has been documented that cancer cells rely on glycolysis for energy production instead of oxidative phosphorylation, a more efficient process of producing ATP [4]. Amino acid uptake, steady-state levels, and catabolism are all elevated in leukemia stem cell population. Still, they depend on oxidative phosphorylation and have lower glycolytic reserves than mature cancer cells [30]. Jones et al. demonstrate that leukemia stem cells isolated from acute myeloid leukemia patients uniquely rely on amino acid metabolism for oxidative phosphorylation and survival. Accordingly, inhibition of amino acid metabolism reduces oxidative phosphorylation and induces cell death [31]. Here, we used different cancer cell models to demonstrate the specific anti-leukemic effects of a novel l-lysine-depleting enzyme from fungus. The l-lysine α-oxidase (LO) from *Trichoderma harzianum* is a flavoprotein from the family of l-amino acid oxidases (LAAOs) [32], an enzyme family extensively studied in medicine for their cytotoxic effects in different tumor cells. However, the large-scale expression and isolation in prokaryotic hosts such as *E. coli* [17] is a challenge for the development of commercial recombinant LAAOs, mainly due to the formation of inclusion bodies [33], production of the inactive [34], and/or low yield [35]. Another issue with the heterologous expression of these enzymes is that they are usually expressed as propeptide precursors to protect the host organism from the LAAO toxicity [35]. We employed several strategies to circumvent these challenges, such as adding the coenzyme FAD and lowering the temperature following induction of heterologous protein expression. The use of low temperatures in bacterial culture reduces system free energy and, therefore, protein synthesis rates, which favors the correct folding [36].

LO gene was succussed cloned into the pGEX-4T1 expression vector, with a N-terminal glutathione S-transferase (GST) tag, which is known to increase protein solubility [37]. Furthermore, as denaturing conditions are not necessary for any purification step, the expressed protein is more likely to maintain its structure and function. In most cases, the presence of GST does not affect its activity or antigenicity [38]. Belviso et al. produced GST-fused asparaginase and tested the enzyme's toxicity in K562, NALM-6, and MOLT-4 leukemic cells. Meanwhile, the fused enzyme showed high toxicity, the GST control had no impact on cell death [39]. Here, we showed that GST alone had no significant effect on cell viability nor in the promotion of apoptotic events, i.e., the toxicity of rLO can only be attributed to rLO activity. However, the fusion with GST was important to the catalytic activity of rLO, since without it (rLO^GST−^) the cytotoxic effects were not observed.

Strikingly, rLO was able to promote cell death by necrosis in CD34 + hematopoietic cells of leukemia patients, but healthy donor cells showed neither apoptosis nor necrosis following rLO treatment. Eradicating the malignant stem cell pool is the ultimate challenge in treating leukemia. Leukemic stem cells (LSC) hijack the normal hemopoietic niche by increasing the expression of efflux pumps, promoting quiescence [40] and the protection provided by the bone marrow microenvironment [41]. Michelozzi et al. explored the susceptibility of leukemia stem cells to ASNase (L-asparaginase), which was effective against CD34 + /CD38 + and CD34 + /CD38- bone marrow cells [42].

The antiproliferative effect of rLO was evaluated in the leukemic cell line Jurkat through the PDL assay. Jurkat cells are susceptible to rLO treatment, with doses higher than 2 mU/mL promoting a significant reduction in the cell viability. Treatment with 10 mU/mL rLO rendered the cells completely unviable after 48 h, preventing the progress of the assay. Our results corroborate with the study of Zhukova et al. [43], in which the authors analyzed the effect of LO on the Burkitt lymphoma cells cycle in more detail, showing that 1 mU/mL LO prevented the transition from S to G2/M phase, when DNA replication occurs. Several studies demonstrate the role of LAAOs in inducing apoptosis, necrosis, and autophagy [44], but their mechanisms of action and the signaling pathways involved remain elusive. In one of the few works on the matter, Pontes et al. treated neutrophils with *Calloselasma rhodosthoma*’s LAAO, which lead to cell death and the activation of the p38 MAPK and PI3K pathways [45]. Here, we also evaluated several pathways related to cell death after rLO treatment: JNK, ERK, p38, PKC, and PTK. In line with Pontes et al. we found that rLO-promoted cell death depends on the activation of JNK and p38 since rLO effects were blunted by inhibitors of these pathways [45]. JNK and p38 are members of the MAPK family of serine/threonine and tyrosine kinases that regulate diverse cellular activities related to cancer development, including proliferation, differentiation, apoptosis, autophagy, and inflammation [46]. In particular, JNK (N-terminal c-Jun kinase) and p38 pathways are proapoptotic in healthy cells, and failure to activate these cascades is strongly involved in carcinogenesis [47].

Although not an l-amino acid oxidase, l-asparaginase promotes amino acid depletion and is the closest example to rLO in the market. l-asparaginase has been used for over 40 years to treat ALL and its mechanism of action has been well-characterized. Studies report that the ERK and Akt/mTOR are the main signaling pathways involved in l-asparaginase-induced apoptosis and autophagy [48]. The first study to evaluate the antitumor effect of LO was published in 1979 by Kusakabe et al. in which they observed that LO inhibits the growth of L5178Y mouse leukemic cells at low doses in vitro. At a 1 mU/mL LO inhibited cell growth in 50% (IC50), and complete inhibition was achieved with 3.3 mU/mL [49]. Besides, an in vitro study with radioactive isotopes showed that L5178Y leukemia cells, CaOv human ovarian carcinoma cells, and Burkitt lymphoma cells almost completely suppressed the DNA synthesis after being incubated for 40 min with LO [50]. The synthesis of RNA and proteins is also affected, with inhibition of approximately 70% [18].

Furthermore, LO from *Trichoderma* cf. *aureoviride Rifai* presented cytotoxicity in low concentrations against the following cell lines: K562, LS174T, HT29, SCOV3, PC3, and MCF7 with the IC50 ranging from 3.0 × 10^–3^ to 0.78 × 10 mU/mL [51]. Two events are supposed to be involved in LO cytotoxicity: the depletion of l-lysine in the medium and the production of hydrogen peroxide. Some authors defend that hydrogen peroxide may be considered the primary mechanism of LO cytotoxic effect on neoplastic cells in vitro. They propose that the DNA breakage observed in cancer cells treated with LO is probably due to oxidative stress promoted by H_2_O_2_, since the pre-incubation with catalase partially prevented the cell growth inhibition effect promoted by LO [50]. On the other hand, other studies show that the growth-inhibitory effect are, at least in part, caused by the decrease of l-lysine concentration in the culture medium. The l-lysine concentration is undetectable after 2 h of incubation of LO and after replenishment of l-lysine, cell growth that was previously inhibited is restored, indicating that l-lysine depletion plays an important role in the cytotoxic effect of LO. Thus, it is unclear if hydrogen peroxide or amino acid depletion contributes most to the inhibition effect, and more studies must be done. The other sub-products of the enzymatic reaction, delta-piperidine-2-carboxylate, and ammonia could not promote inhibition in cell growth in vitro [49].

**Fig. 7:**
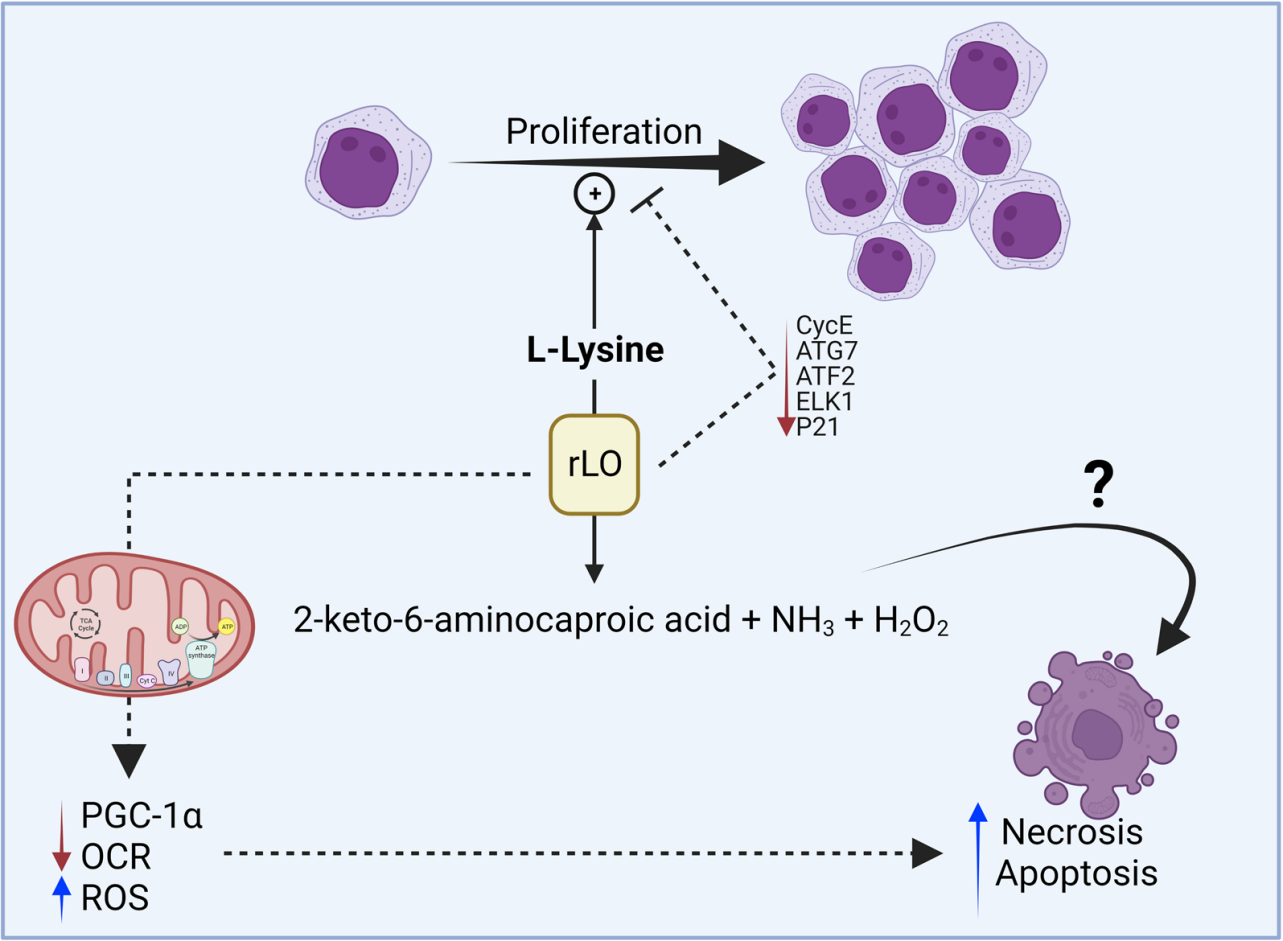
Possible mechanism of rLO action in Jukart cells. l-lysine is an essential amino acid for Jurkat cell proliferation. The degradation of l-lysine by rLO promotes the inhibition of cell proliferation by decreasing the expression of genes from the JNK and p38 pathways. Furthermore, the absence of l-lysine leads to an increase in ROS and decreases in OCR and PGC-1α expression

The metabolic rewiring in cancer to support faster growth rates creates a window of intervention that differentiates normal and leukemic cells. This rewiring includes, for example, the glycolytic shift and alternative anaplerotic pathways [52]. Mitochondria are at the core of this adaptation, and the combination of mitochondrial-targeting drugs and radiotherapy has been proposed as a therapeutic strategy [53]. In this context, the reduction of mitochondrial function and triggering of apoptotic pathways, centered in the release of cytochrome c following the decrease in mitochondrial membrane potential, are mediated by p38 phosphorylation and Bax accumulation in the mitochondria [54, 55]. Indeed, evasive mechanisms that can maintain mitochondrial function are mediated by the master regulator of mitochondrial biogenesis PGC-1α [56, 57] (Fig. 7). In line with these findings, we demonstrated that rLO treatment impairs mitochondrial oxidative capacity and decreases PGC-1α expression in Jurkat cells, suggesting a potential accessory clinical intervention. The increase in mitochondrial superoxide production further supports an impairment of mitochondrial integrity, which could be an effect of rLO-produced peroxide. rLO is inactive after 4 h in culture conditions (data not shown). Therefore, the increase in hydrogen peroxide measured after 24 h of treatment is due to the surge in cell-generated reactive oxygen species rather than a direct measurement of the enzymatic product. Future work to dissect which outcome of rLO reaction is the major cause of the reduction in cell viability may help determine synergic drugs.

## Conclusions

In conclusion, this report highlights the effects of treating leukemic cells with recombinant *T. harzianum* l-lysine α-oxidase, gathering evidence to support future translational studies. rLO treatment induces both cell death and cell cycle stasis, effects that are dependent on apoptotic pathways and that could be triggered, at least partially, by mitochondrial dysfunction.

## Methods

### Native LO production

*T. harzianum* ALL42 strain was maintained in PDA (Potato Dextrose Agar) medium at 4 °C. ALL42 was grown on PDA plates at 25 °C for seven days to complete sporulation. To produce LO, the conidia were pre-cultivated by semi-solid-state fermentation on 4 g wheat bran supplemented with 0.9% NaNO_3_ solution and 2.5 mL distilled water in a 125 mL Erlenmeyer at 28 °C for seven days [58]. The inoculum was then transferred to 500 mL Erlenmeyer containing 20 g wheat bran supplemented with 0.9% NaNO_3_ and 10 mL distilled water at 28 °C for 15 days. Proteins were harvested from the medium by adding 100 mL of distilled water to the flask, which was kept stirring at 28 °C and 150 rpm for 2 h. The samples were filtrated and stored at − 20 °C until enzymatic activity and purification steps were performed [59].

### Purification of native LO

Filtered culture media was subjected to two chromatographic steps using an FPLC system (ÄKTA FPLC™ Fast Protein Liquid Chromatograph, GE Healthcare) to isolate native LO. A DEAE Sephadex FF anion (1 × 40 cm) exchange column was previously equilibrated with 25 mM Tris–HCl buffer pH 7.5 was used in the first step. Fractions identified to have enzymatic activity (described in the next section) were concentrated using Amicon^®^ Centrifugal Filters (Merck) and submitted to the next purification step. These fractions were further purified by gel filtration using a Sephacryl S200-HR column (0.45 × 95 cm), previously equilibrated with 20 mM ammonium bicarbonate buffer pH 7.5. The collected fractions were tested for enzymatic activity and subjected to electrophoretic characterization in SDS-PAGE gel. Protein concentration was determined using the Bradford assay using bovine serum albumin (BSA) as a standard.

### LO activity assay

LO activity was assayed according to Kusakabe [58] adapted to a microplate format. The standard reaction mixture consisted of 10 µL of 100 mM l-lysine, 35 µL of 200 mM potassium phosphate buffer pH 8.0, 10 µL of 1 U/mL catalase, and 35 μL of deionized water. The mixture was maintained at 37 °C for 2 min, and 10 μL of the enzyme LO was added. The tube was incubated at 37 °C for 20 min with shaking. After this time, the reaction was interrupted by adding 20 µL of 25% trichloroacetic acid. The tube was centrifuged at 6000×*g* for 5 min. A volume of 30 μL of the supernatant was transferred to a thermal microplate with 30 μL of 0,1% w/v 3-methyl-2-benzothiazolinone hydrazone and 70 μL of 1 M sodium acetate buffer pH 5 and 70 μL of deionized water. The plate was incubated at 50 °C for exactly 30 min. The α-keto acid formed was determined in a spectrophotometer (xMarkTM, Bio-Rad) at 317 nm. One unit of the enzyme was defined as the amount of enzyme that catalyzes the formation of 1 μmol of 6-amino-2-oxohexanoic acid from L-lysine per minute at 37 °C and pH 8.

### Protein identification by mass spectrometry

The confirmation of the LO amino acid sequence was determined by mass spectrometer Xevo TQ-S Triple Quadrupole coupled to ACQUITY UPLC in the C18 column (Waters) using the partially purified sample. Samples were reduced, alkylated, and digested with trypsin. Peptides were identified using the TPP data search pipeline [59] and the *T. harzianum* protein sequences from the genomic data bank. (http://genome.jgi.doe.gov/Triha1/Triha1.home.html).

### LO cloning

By aligning *T. harzianum* LAAO coding sequences in the NCBI database (GenBank), specific primers were designed to amplify the gene cDNA (forward, 5′-GGGGGGATCCGACAATGTTGACTTTG-3′; reverse, 5′-GGGGGAATTCTTAGACCTTCACCTGG-3′). Reactions were performed in a T100™ Thermal Cycler (Bio-Rad). Amplification was performed at 95 °C for 2 min, followed by 35 cycles at 95 ºC for 1 min, 58 °C for 1.5 min, 72 °C for 2 min, and one cycle at 72 °C for 10 min. The resulting amplicons were cloned in pGEX-4T1 vector (GE Healthcare) using *BamHI* and *EcoRI* (FastDigest, Fermentas) restriction sites, and the ligation product was transformed into E. *coli* DH5α. The bacteria were grown in an LB medium containing 100 µg/mL Ampicillin at 37 °C. The vector map is present in Additional file 1: Fig. S2.

### Heterologous expression

*E. coli* BL21(DE3) was used as a host for heterologous expression. The bacteria were pre-inoculated in 4 mL LB medium containing 100 µg/mL Ampicillin at 37 °C overnight and then diluted in 100 mL LB medium in the same conditions when the absorbance at 600 nm reached 0.6 0.5 mM isopropyl-β-D-thiogalactopyranoside (IPTG) and 50 µM flavin adenine dinucleotide (FAD) were added to the medium. After 12 h, cells were harvested by centrifugation for 20 min at 10,000*g*. The pelleted was resuspended in TTBS buffer (50 mM Tri-HCl pH 7.2, 0.1 M NaCl, and 0.05% Tween 20) containing 10 U lysozyme, 1 mM phenylmethanesulfonylfluoride fluoride (PMSF) and 20% Triton X-100. Cells were disrupted in sonication using an ultrasonic generator (Fisherbrand, model 120), and the soluble proteins were collected by centrifugation at 20,000×*g* for 20 min. To test the effects of the tag on cell viability, GST was produced by transforming empty pGEX-4T1 vector into BL21 cells. The same procedures described above for the full protein were followed, except for the temperature shift and FAD supplementation.

### Recombinant LO purification

The GST-tagged proteins were purified using a Glutathione Sepharose 4 Fast Flow affinity column (GE Healthcare) (Additional file 1: Fig. S3). The proteins were applied in a column in 0.1 M Tris–HCl pH 7.4 buffer and eluted with 0.1 M reduced glutathione. Purification efficiency was analyzed by 12% sodium dodecyl sulfate–polyacrylamide gel electrophoresis (SDS-PAGE) following the Laemmli protocol. Proteins were visualized by staining with Coomassie Blue R-250. Purified proteins were incubated with Glutathione Thrombin Protease (Novagen) for removal of the GST moiety. The reaction was realized in the presence of Glutathione Sepharose resin for GST removal. The protein samples were stored in 50% glycerol at – 20 ºC.

### Enzyme characterization of recombinant and native LO

To determine the optimal pH for LO, different assay buffers were employed: 100 mM HCl/KCl pH 2, 100 mM citrate/phosphate (pH 3.0, 4.0, 5.0, 6.0, and 7.0), 100 mM phosphate (pH 7.5 and 8.0), 100 mM glycine (pH 8.0, 8.6, 9.0 and 10.0). The optimal temperature was determined by assaying at 4, 10, 20, 25, 37, 45, 50, 60, 80, and 100 °C. The effect of incubation time on enzyme activity was determined at 10, 20, 30, and 40 min. Catalytic specificity was determined by testing different amino acids as substrates. The following kinetic parameters were determined using SigrafW 2.0 software: Michaelis–Menten constant (K_M_), maximum velocity (V_max)_, turnover number (K_cat_), specificity constant (kcat/K_M_), and Hill number (n_H_) (Additional file 1: Fig. S4 and Tables S3, S4).

### Cell cultures, healthy volunteers, and treatments

Jurkat E6.1 human acute leukemia T cell line was grown in advanced Roswell Park Memorial Institute (RPMI) 1640 medium (Gibco®, Life Technologies, Carlsbad, CA, USA) supplemented with 10% fetal bovine serum, 2 mM l-glutamine, 2500 mg/L glucose, 10 mM HEPES, 100 U/mL penicillin and 100 μg/mL streptomycin in a humidified 5% CO_2_ atmosphere at 37 °C. Cell pellets were obtained by centrifugation at 300×*g* for 5 min at 4 °C. The cell concentration was maintained as recommended by the ATCC cell biology collection.

Peripheral blood mononuclear cells (PBMC) collected from healthy volunteers were used as controls. To obtain the PBMC, peripheral blood from healthy donors was collected in heparinized syringes (Hepamax S, Blausiegel) and centrifuged at 160×*g* for 20 min at room temperature. Plasma was collected and submitted to new centrifugation at 1100×*g* for 10 min at room temperature. The platelet-containing plasma pellet was discarded, and the supernatant was returned to the first pellet. PBMC were separated from neutrophils by centrifugation at 450×*g* for 50 min, in a Mono-Poly Resolving Medium gradient (MP Biomedicals), according to the manufacturer’s instructions. The PBMCs obtained were washed with RPMI medium supplemented with 10% FBS to remove Ficoll. The number of cells was determined in a Neubauer chamber. This study was approved by the local Research Ethics Committee (CAAE number 15863019.9.0000.5440), and all volunteers gave their written informed consent before blood sample collection. This study is in accordance with the Declaration of Helsinki.

CD34 + cells were isolated from PBMC using anti-CD34 magnetic beads (MicroBead Kit, Miltenye Biotec) according to the manufacturer’s instructions. Briefly, the labeled cell suspension was loaded into a MACS column and placed in a magnetic field to retain the magnetically labeled CD34 + cells, which were eluted after removing the column from the magnetic field.

### Flow cytometry analysis

Jurkat and PBMCs (both at 1.10^5^ cells/mL) were treated with rLO (1–10 mU/mL) or ATO (1.5 µM) and maintained for 24 h at 37 °C in a humidified incubator with 5% of CO_2_. Then, the cells were stained with Annexin V-FITC (BD Biosciences, cat. nº65874X) for 30 min following the manufacturer’s instructions and 1 µg/mL propidium iodide (PI) for an additional 5 min. ATO-treated cells were stained with Annexin V only or PI only to shape the gates of double-positive cells, and untreated cells were used to define double-negative populations. The proportion of dead (double stained) and apoptotic (Annexin V + /PI−) cells was assessed by FITC and PI fluorescence intensity using flow cytometry (Guava EasyCyte™ Mini System).

### Time-lapse microscopy

The binding of Annexin V-FITC and incorporation of PI in Jurkat cells treated with rLO were also analyzed by time-lapse fluorescence microscopy to observe the rLO’s action time. For this, 250 µL at 4 × 10^6^ cell/mL Jurkat cells were treated with 1 mU/mL of rLO in RPMI medium and immediately incubated with 7 μL of Annexin V-FITC (BD Biosciences, ex/em 494/517 nm) and 2 μL of PI 1 μg/mL (Sigma- Aldrich, ex/em 493/636 nm). The cells were transferred to a glass-bottom optical plate (MatTek Corporation) and imaged with a microscope equipped with an incubator chamber to maintain 37 °C and 5% CO_2_ concentration (BioStation IMq, Nikon—Laboratory of Biophotonic Image FMRP/USP). Image capture started after 1 h to stabilize the culture and adjust the focus. Eight fields were selected, and a photo was taken every 3 min. After 18 h, all the images were gathered to produce a video that allowed the observation of the Annexin-V binding and PI incorporation by Jurkat cells over time. Untreated cells were used as a control.

### Population doubling level (PDL) assay

K562 and Jurkat cells were distributed in 96-well microplates (1 × 10^5^ cell/mL) and incubated for 48 h at 37 ºC with rLO (0.1 mU/mL, 0.2 mU/mL, 0.5 mU/mL, 1 mU/mL, 2 mU/mL and 10 mU/mL), ATO (1.5 mM), or medium alone. After 48 h of the treatment, cell density was determined by counting in a Neubauer chamber. These cells were inoculated in a new subculture, which was maintained under the same initial conditions (1 × 10^5^ cells/mL) and treated with rLO again. After 144 h, it was checked whether the treatment could inhibit the growth of cancer cells. The cumulative PDL was determined for each condition, and the following equation was used: 2 × [log(Cf/Ci)] + PDLp. The PDL was calculated every 48 h by considering the following parameters: Cf: final cell concentration; Ci: initial cell concentration (1 × 10^5^ cells/mL); and PDLp: previous PDL.

### Treatment of Jurkat cells with cell signaling inhibitors

Jurkat cells (1 × 10^4^ cells/mL) were distributed in 96-well microplates and pre-treated with the following pharmacological inhibitors at 20 µM: Genistein (PTKs inhibitor), PD98059 (ERK inhibitor), SB202190 (p38 MAPK inhibitor), H-7 (PKC inhibitor), and SP600125 (JNK inhibitor), all purchased from Sigma-Aldrich. After 3 h of incubation, the Jurkat cells were treated for 24 h with 1 mU/mL rLO or medium alone. Jurkat cells were analyzed by flow cytometry to determine Annexin V-FITC binding and PI incorporation. The results were expressed as the frequency of PI negative, Annexin V/PI positive, or Annexin V positive stained cells as a percentage value.

### Oxygen consumption assays

Jurkat cells were treated as described elsewhere and collected for oxygen consumption assays in a High-Resolution Oxygraph (Oroboros O2K, Oroboros, Innsbruck, Austria). Cells were harvested by centrifugation, and 3 × 10^6^ cells were loaded into respirometer chambers containing pre-warmed cell culture media. Respirometry parameters were verified by the sequential addition of drugs affecting the mitochondrial electron transfer chain, as follows: 1 µM oligomycin, carbonyl cyanide 3-chlorophenylhydrazone was titrated until maximal oxygen consumption rate (OCR) was reached, and finally, 2 µM antimycin was added to obtain non-mitochondrial respiration, which was subtracted of all values before calculation. Spare capacity was calculated by subtracting the initial OCR of maximal OCR. Proton leak was considered as the OCR after oligomycin addition, and ATP-linked OCR was the difference between the later and initial OCR.

### Reactive oxygen species assay

Cells were incubated for 30 min with MitoSOX (Thermo) mitochondrial superoxide fluorescent probe in complete culture media, washed with Krebs–Henseleit buffer, and resuspended in the same buffer for fluorescence measurements ex/em. 510/580 nm. For peroxide production measurement, the cells were initially incubated for 15 min with Amplex™ UltraRed Reagent in Krebs–Henseleit buffer, and then fluorescence signal at ex/em. 530/590 nm was measured every 10 min for 1 h. All assays were normalized by cell number.

### Gene expression analysis

Jurkat cells were treated as indicated and harvested by centrifugation. Total RNA was extracted using TRIzol™ (Invitrogen) following the manufacturer's instructions, and 2 μg was used for cDNA synthesis using the High-Capacity Reverse Transcription Kit. qPCR reactions were performed with SYBER Green Master Mix (Thermo) using the primers described in Additional file 1: Table S5. The relative gene expression was calculated using the Livaak method normalized by RPL39 housekeeping and displayed as fold-change over the control.

### Statistical analysis

The results are presented as means ± standard deviation of the mean. All data were analyzed using Prism (GraphPad Prism 8 Software, la Jolla, CA, USA). The statistical differences among group means were determined by one-way analysis of variance (1-way ANOVA) followed by Bonferroni´s multiple comparison test or Student’s T-test. Differences that provided p < 0.05 were considered statistically significant.

### Supplementary Information

**Additional file 1: Figure S1.** Purification of native rLO. **A** Step 1: Ion exchange chromatography. The native LO protein was purified based on its isoelectric point by the DEAE Sephadex column for cation exchange chromatography. The graph shows the enzyme activity (o) and protein profile () of the fractions collected through the salt gradient. **B** Step 2: Size exclusion chromatography. The LO protein was selected from its molecular size in a Sephacryl S-200 HR column. The graph shows the enzyme activity profile and the protein content of the fractions collected. The blue line shows the LO activity and the red line shows the absorbance at 280 nm. **Figure S2.** pGEX-4T1-LO vector map. **A** The pGEX-4T1 bacterial vector is used to express GST-fused proteins. The vector has a multiple cloning region that contains several restriction sites. In orange: LO gene was added between BamHI and EcoRI restriction sites. Between the GST tag and the cloning sites, there is a thrombin cleaving site that allows GST removal. It also has a lac promoter for induction with IPTG and a gene that attributes resistance to ampicillin. **B** GST-rLO DNA sequence cloned into a pGEX-4T1 vector with GST highlighted in blue and rLO in green. **Figure S3.** Purification of rLO. **A** Chromatographic profile of rLO purification by affinity chromatography. In blue: enzymatic activity of the fractions collected. In red: Absorbance of the samples at 280 nm. The firsts fractions The first fractions correspond to the crude extract. After washing the column, the enzyme activity falls and rises again when the elution of rLO begins. **Figure S4.** Enzymatic characterization of rLO. **A** Effect of pH on rLO in percentage of relative activity. The tested pHs were: 2, 3, 4, 5, 6, 7, 7.5, 8, 8.6, 9, and 10 with different buffers. **B** Effect of temperature on rLO activity. The incubation temperatures tested were 4, 10, 20, 25, 37, 50, 60, 70, 80, and 100 ^o^C. **C** Effect of substrate concentration on rLO activity. The concentrations used were: 0.1, 0.5, 1, 5, 10, 50, 100, 200, and 300 mM of l-lysine. **D** Graph of substrate concentration versus initial velocity (V0) to determine the Michaelis-Menten constant (K_M_/K_0.5_) and Maximum velocity (V_máx_). **Figure S5.** Comparison of the effect in the viability of rLO, rLO^GST-^ and GST in Jurkat cells. GST-tagged rLO (rLO) was produced in *E. coli* with pGEX-4T1-LO vector and the untagged protein (rLO^GST-^) was obtained using a thrombin cleavage site between GST and rLO. GST was produced in *E. coli* transformed with pGEX-4T1 empty vector. ATO was used as a positive control. **A**, **D** Effect of treatments on cell viability. **B**, **E** Percentage of positive cells for Annexin V only (cells in apoptosis). **C**, **F** Percentage of positive cells in late apoptosis (double marked for Annexin V and PI). All treatments were carried out with enzymes at a concentration of 1 mU/mL. **Table S1.** Purification yield of native LO. **Table S2.** Mass spectrometry analyses and blast p results. **Table S3.** Substrate specificity data of rLO for different l-amino acids. **Table S4.** Kinetic parameters were calculated for rLO with l-lysine as substrate. **Table S5. **List of signaling pathways, primers, and genes evaluated by qPCR.

## Data Availability

Not applicable.

## References

[1] Hanahan D, Weinberg RA (2000). The hallmarks of cancer. Cell.

[2] Hanahan D, Weinberg RA (2011). Hallmarks of cancer: the next generation. Cell.

[3] Hsu PP, Sabatini DM (2008). Cancer cell metabolism: warburg and beyond. Cell.

[4] Warburg O (1956). On the origin of cancer cells. Science.

[5] Fung MKL, Chan GCF (2017). Drug-induced amino acid deprivation as strategy for cancer therapy. J Hematol Oncol.

[6] Lukey MJ, Katt WP, Cerione RA (2017). Targeting amino acid metabolism for cancer therapy. Drug Discov Today..

[7] Chen R, Zou Y, Mao D, Sun D, Gao G, Shi J (2014). The general amino acid control pathway regulates mTOR and autophagy during serum/glutamine starvation. J Cell Biol.

[8] Onodera J, Ohsumi Y (2005). Autophagy is required for maintenance of amino acid levels and protein synthesis under nitrogen starvation. J Biol Chem.

[9] Martinet W, De Meyer GRY, Herman AG, Kockx MM (2005). Amino acid deprivation induces both apoptosis and autophagy in murine C2C12 muscle cells. Biotechnol Lett.

[10] Pokrovsky VS, Chepikova OE, Davydov DZ, Zamyatnin AA, Lukashev AN, Lukasheva EV (2017). Amino acid degrading enzymes and their application in cancer therapy. Curr Med Chem.

[11] Kawedia JD, Rytting ME (2014). Asparaginase in acute lymphoblastic leukemia. Clin Lymphoma Myeloma Leuk.

[12] Vander Heiden MG (2011). Targeting cancer metabolism: a therapeutic window opens. Nat Rev Drug Discov.

[13] Lieu EL, Nguyen T, Rhyne S, Kim J (2020). Amino acids in cancer. Exp Mol Med.

[14] Kocher RA (1944). Effects of a low lysine diet on the growth of spontaneous mammary tumors in mice and on the N2 balance in man. Cancer Res.

[15] Kocher RA (1915). The hexone bases of malignant tumors. J Biol Chem.

[16] Reiken SR, Briedis DM (1992). The effect of lysine deprivation on leukemic blood. Amino Acids.

[17] Pollegioni L, Motta P, Molla G (2013). L-Amino acid oxidase as biocatalyst: a dream too far?. Appl Microbiol Biotechnol.

[18] Pokrovsky VS, Treshalina HM, Lukasheva EV, Sedakova LA, Medentzev AG, Arinbasarova AY (2013). Enzymatic properties and anticancer activity of l-lysine α-oxidase from *Trichoderma* cf. *aureoviride* Rifai BKMF-4268D. Anticancer Drugs.

[19] Silva RN, Monteiro VN, Steindorff AS, Gomes EV, Noronha EF, Ulhoa CJ (2019). Trichoderma/pathogen/plant interaction in pre-harvest food security. Fungal Biol.

[20] Costa MN, Silva RN (2022). Cytotoxic activity of l-lysine alpha-oxidase against leukemia cells. Semin Cancer Biol.

[21] Smirnova IP, Karimova EV, Shneider YA (2017). Antibacterial activity of l-lysine-α-oxidase from the *Trichoderma*. Bull Exp Biol Med.

[22] Wong CH, Iskandar KB, Yadav SK, Hirpara JL, Loh T, Pervaiz S (2010). Simultaneous induction of non-canonical autophagy and apoptosis in cancer cells by ROS-dependent ERK and JNK activation. PLoS ONE.

[23] Plotnikov A, Zehorai E, Procaccia S, Seger R (2011). The MAPK cascades: signaling components, nuclear roles and mechanisms of nuclear translocation. Biochim Biophys Acta Mol Cell Res.

[24] Kirsch K, Zeke A, Tőke O, Sok P, Sethi A, Sebő A (2020). Co-regulation of the transcription controlling ATF2 phosphoswitch by JNK and p38. Nat Commun.

[25] Van Loo G, Saelens X, Van Gurp M, MacFarlane M, Martin SJ, Vandenabeele P (2002). The role of mitochondrial factors in apoptosis: a Russian roulette with more than one bullet. Cell Death Differ.

[26] Puigserver P, Spiegelman BM (2003). Peroxisome proliferator-activated receptor-gamma coactivator 1α (PGC-1α): transcriptional coactivator and metabolic regulator. Endocr Rev.

[27] Vazquez F, Lim JH, Chim H, Bhalla K, Girnun G, Pierce K (2013). PGC1α expression defines a subset of human melanoma tumors with increased mitochondrial capacity and resistance to oxidative stress. Cancer Cell.

[28] Nakamura H, Takada K (2021). Reactive oxygen species in cancer: current findings and future directions. Cancer Sci.

[29] Perillo B, Di Donato M, Pezone A, Di Zazzo E, Giovannelli P, Galasso G (2020). ROS in cancer therapy: the bright side of the moon. Exp Mol Med.

[30] Lagadinou ED, Sach A, Callahan K, Rossi RM, Neering SJ, Minhajuddin M (2013). BCL-2 inhibition targets oxidative phosphorylation and selectively eradicates quiescent human leukemia stem cells. Cell Stem Cell.

[31] Jones CL, Stevens BM, D’Alessandro A, Reisz JA, Culp-Hill R, Nemkov T (2018). Inhibition of amino acid metabolism selectively targets human leukemia stem cells. Cancer Cell.

[32] Edakova LU, Irsova GA (2000). Anticancer enzyme l-lysine α-oxidase. Appl Biochem Biotechnol.

[33] Nuutinen JT, Marttinen E, Soliymani R, Hilden K, Timonen AS (2012). l-Amino acid oxidase of the fungus *Hebeloma cylindrosporum* displays substrate preference towards glutamate. Microbiology.

[34] Kitagawa M, Ito N, Matsumoto Y, Saito M, Tamura T, Kusakabe H (2021). Structural basis of enzyme activity regulation by the propeptide of l-lysine α-oxidase precursor from *Trichoderma viride*. J Struct Biol X.

[35] Fakruddin M, Mohammad Mazumdar R, Bin Mannan KS, Chowdhury A, Hossain MN (2013). Critical factors affecting the success of cloning, expression, and mass production of enzymes by recombinant *E. coli*. ISRN Biotechnol.

[36] Rosemberg IM (2007). Protein analysis and purification: benchtop techniques.

[37] Esposito D, Chatterjee DK (2006). Enhancement of soluble protein expression through the use of fusion tags. Curr Opin Biotechnol.

[38] Tani Y, Omatsu K, Saito S, Miyake R, Kawabata H, Ueda M (2015). Heterologous expression of l-lysine α-oxidase from *Scomber japonicus* in *Pichia pastoris* and functional characterization of the recombinant enzyme. J Biochem.

[39] Belviso S, Iuliano R, Amato R, Perrotti N, Menniti M (2017). The human asparaginase enzyme (ASPG) inhibits growth in leukemic cells. PLoS ONE.

[40] Siveen KS, Uddin S, Mohammad RM (2017). Targeting acute myeloid leukemia stem cell signaling by natural products. Mol Cancer.

[41] Korn C, Méndez-Ferrer S (2017). Myeloid malignancies and the microenvironment. Blood.

[42] Michelozzi IM, Granata V, De Ponti G, Alberti G, Tomasoni C, Antolini L (2019). Acute myeloid leukaemia niche regulates response to l-asparaginase. Br J Haematol.

[43] Zhukova OS, Khaduev SK, Dobrynin IV, Smirnova MP, Lukasheva EV (1985). Effect of l-lysine-alpha-oxidase on the cell cycle kinetics of cultured Burkitt’s lymphoma cells. Eksp Onkol.

[44] Ande SR, Kommoju PR, Draxl S, Murkovic M, MacHeroux P, Ghisla S (2006). Mechanisms of cell death induction by l-amino acid oxidase, a major component of ophidian venom. Apoptosis.

[45] Pontes AS, Setúbal SDS, Nery NM, Da Silva FS, Da Silva SD, Fernandes CFC (2016). P38 MAPK is involved in human neutrophil chemotaxis induced by l-amino acid oxidase from *Calloselasma rhodosthoma*. Toxicon.

[46] Kim EK, Choi EJ (2015). Compromised MAPK signaling in human diseases: an update. Arch Toxicol.

[47] Peluso I, Yarla NS, Ambra R, Pastore G, Perry G (2019). MAPK signalling pathway in cancers: Olive products as cancer preventive and therapeutic agents. Semin Cancer Biol.

[48] Song P, Wang Z, Zhang X, Fan J, Li Y, Chen Q (2017). The role of autophagy in asparaginase-induced immune suppression of macrophages. Cell Death Dis.

[49] Kusakabe H, Kodama K, Kuninaka A, Yoshino H, Soda K (1980). Effect of l-lysine α-oxidase on growth of mouse leukemic cells. Agric Biol Chem.

[50] Khaduev SK, Zhukova OS, Dobrynin IV, Soda K, Berezov TT (1986). Comparative study of the effect of l-lysine-l-oxidase from *Trichoderma harzianum* Rifai and *Trichoderma viride* on nucleic acid synthesis in human tumor cells in vitro. Biull Eksp Biol Med.

[51] Pokrovsky VS, Lukashev AN, Babayeva G, Karshieva SS, Arinbasarova AY, Medentzev AG (2021). Plasma pharmacokinetics and tissue distribution of L-lysine α-oxidase from *Trichoderma* cf. *aureoviride* RIFAI VKM F- 4268D in mice. Amino Acids.

[52] Shiratori R, Furuichi K, Yamaguchi M, Miyazaki N, Aoki H, Chibana H (2019). Glycolytic suppression dramatically changes the intracellular metabolic profile of multiple cancer cell lines in a mitochondrial metabolism-dependent manner. Sci Rep.

[53] Liu Y, Shi Y (2020). Mitochondria as a target in cancer treatment. MedComm.

[54] Kang YH, Lee SJ (2008). The role of p38 MAPK and JNK in arsenic trioxide-induced mitochondrial cell death in human cervical cancer cells. J Cell Physiol.

[55] Mandal C, Dutta A, Mallick A, Chandra S, Misra L, Sangwan RS (2008). Withaferin A induces apoptosis by activating p38 mitogen-activated protein kinase signaling cascade in leukemic cells of lymphoid and myeloid origin through mitochondrial death cascade. Apoptosis.

[56] Chaudhary S, Ganguly S, Palanichamy JK, Singh A, Bakhshi R, Jain A (2021). PGC1A driven enhanced mitochondrial DNA copy number predicts outcome in pediatric acute myeloid leukemia. Mitochondrion.

[57] Sarry JE, Recher C, Aroua N (2018). Extracellular ATP and CD39 regulates mitochondrial function and cytarabine resistance through intrinsic PKA-ATF-PGC1a pathway in acute myeloid leukemia. Blood.

[58] Kusakabe H, Kodama K, Kuninaka A, Yoshino H, Misono H, Soda K (1980). A new antitumor enzyme, l-lysine a-oxidase from *Trichoderma viride*. J Biol Chem.

[59] Deutsch EW, Mendoza L, Shteynberg D, Slagel J, Sun Z, Moritz RL (2015). Trans-Proteomic Pipeline, a standardized data processing pipeline for large-scale reproducible proteomics informatics. Proteom Clin Appl.

