# Supplementary material for: The recombinant l-lysine α-oxidase from the fungus Trichoderma harzianum promotes apoptosis and necrosis of leukemia CD34 + hematopoietic cells
Source: Microb Cell Fact. 2024 Feb 14;23:51. doi: 10.1186/s12934-024-02315-2 (PMC10865671; doi:10.1186/s12934-024-02315-2)
Supplement: Supplementary file 1 — Additional file 1: Figure S1. Purification of native rLO. A Step 1: Ion exchange chromatography. The native LO protein was purified based on its isoelectric point by the DEAE Sephadex column for cation exchange chromatography. The graph shows the enzyme activity (o) and protein profile () of the fractions collected through the salt gradient. B Step 2: Size exclusion chromatography. The LO protein was selected from its molecular size in a Sephacryl S-200 HR column. The graph shows the enzyme activity profile and the protein content of the fractions collected. The blue line shows the LO activity and the red line shows the absorbance at 280 nm. Figure S2. pGEX-4T1-LO vector map. A The pGEX-4T1 bacterial vector is used to express GST-fused proteins. The vector has a multiple cloning region that contains several restriction sites. In orange: LO gene was added between BamHI and EcoRI restriction sites. Between the GST tag and the cloning sites, there is a thrombin cleaving site that allows GST removal. It also has a lac promoter for induction with IPTG and a gene that attributes resistance to ampicillin. B GST-rLO DNA sequence cloned into a pGEX-4T1 vector with GST highlighted in blue and rLO in green. Figure S3. Purification of rLO. A Chromatographic profile of rLO purification by affinity chromatography. In blue: enzymatic activity of the fractions collected. In red: Absorbance of the samples at 280 nm. The firsts fractions The first fractions correspond to the crude extract. After washing the column, the enzyme activity falls and rises again when the elution of rLO begins. Figure S4. Enzymatic characterization of rLO. A Effect of pH on rLO in percentage of relative activity. The tested pHs were: 2, 3, 4, 5, 6, 7, 7.5, 8, 8.6, 9, and 10 with different buffers. B Effect of temperature on rLO activity. The incubation temperatures tested were 4, 10, 20, 25, 37, 50, 60, 70, 80, and 100 oC. C Effect of substrate concentration on rLO activity. The concentrations used were: 0. [file 12934_2024_2315_MOESM1_ESM.docx]

**The recombinant L-Lysine α-oxidase from the fungus *Trichoderma harzianum* promotes apoptosis and necrosis of leukemia CD34+ hematopoietic cells**

Mariana do Nascimento Costa^1^, Thiago Aparecido Silva^2^, Dimitrius Santiago Passos Simões Fróes Guimarães^3^, Rafael Ricci-Azevedo^2^ Felipe Roberti Teixeira^4^, Leonardo Reis Silveira^3^, Marcelo Damário Gomes^1^, Vítor Marcel Faça^1^, Eduardo Brandt de Oliveira^1^, Rodrigo T. Calado^5^, and Roberto N. Silva^1*^

**Additional file**

**Figure S1. Purification of native rLO.** (A) Step 1: Ion exchange chromatography. The native LO protein was purified based on its isoelectric point by the DEAE Sephadex column for cation exchange chromatography. The graph shows the enzyme activity (o) and protein profile () of the fractions collected through the salt gradient. (B) Step 2: Size exclusion chromatography. The LO protein was selected from its molecular size in a Sephacryl S-200 HR column. The graph shows the enzyme activity profile and the protein content of the fractions collected. The blue line shows the LO activity and the red line shows the absorbance at 280 nm.

**Table S1. Purification yield of native LO.**

|  | **LO activity (U/mL)** | **Yield (%)** | **Protein concentration (mg/mL)** | **LO specific activity (U/mg)** |
| --- | --- | --- | --- | --- |
| **Crude extract** | 13.37 | 100 | 0.655 | 20.41 |
| **Ion exchange chromatography** | 7.178 | 53.7 | 0.307 | 23.38 |
| **Size exchange chromatography** | 5.14 | 38.4 | 0.198 | 26.11 |


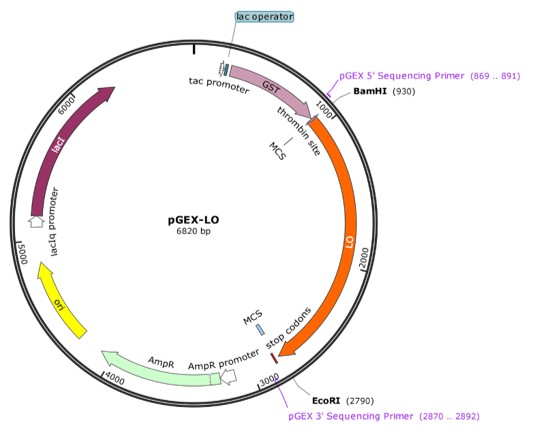


**B.** Cloned rLO sequence

**GST-TAG; rLO**

ATGTCCCCTATACTAGGTTATTGGAAAATTAAGGGCCTTGTGCAACCCACTCGACTTCTTTTGGAATATCTTGAAGAAAAATATGAAGAGCATTTGTATGAGCGCGATGAAGGTGATAAATGGCGAAACAAAAAGTTTGAATTGGGTTTGGAGTTTCCCAATCTTCCTTATTATATTGATGGTGATGTTAAATTAACACAGTCTATGGCCATCATACGTTATATAGCTGACAAGCACAACATGTTGGGTGGTTGTCCAAAAGAGCGTGCAGAGATTTCAATGCTTGAAGGAGCGGTTTTGGATATTAGATACGGTGTTTCGAGAATTGCATATAGTAAAGACTTTGAAACTCTCAAAGTTGATTTTCTTAGCAAGCTACCTGAAATGCTGAAAATGTTCGAAGATCGTTTATGTCATAAAACATATTTAAATGGTGATCATGTAACCCATCCTGACTTCATGTTGTATGACGCTCTTGATGTTGTTTTATACATGGACCCAATGTGCCTGGATGCGTTCCCAAAATTAGTTTGTTTTAAAAAACGTATTGAAGCTATCCCACAAATTGATAAGTACTTGAAATCCAGCAAGTATATAGCATGGCCTTTGCAGGGCTGGCAAGCCACGTTTGGTGGTGGCGACCATCCTCCAAAATCGGATCTGGTTCCGCGTGgatccGACAATGTTGACTTTGCTGAATCTGTCCGAACCCGCTGGGCGAGGCGACTTGTTCGTGAGAAGGTCGCCAAGGAACTCAACATTCTAACCGAAAGACTTGGTGAGGTGCCCGGAATCCCTCCTCCAAACGAAGGCAGGTTCCTGGGCGGCGGCTACTCTCACGACAATCTACCGTCTGATCCCCTCTATTCCAGCATTAAGCCGGCTCCTCTAAAGGAGGCTCCTCGAGCAGAAGAGGAACTGCCGTCTCGAAAGGTGTGCATCGTAGGCGCTGGTGTTTCCGGCCTCTACATAGCCATGATTCTGGATGATTTGAAGATCCCAAACCTCACTTACGACATCTTCGAATCCAGTTCCAGAACTGGTGGCCGTTTGTATACGCACCATTTCACCGACGCCAAGCATGACTATTACGACATTGGTGCTATGCGATACCCTGACATCCCCAGCATGAAACGTACCTTTAACCTGTTTAAACGTACTGGGATGCCTCTCATCAAATATTACCTTGATGGCGAGAATACCCCTCAGCTGTACAATAATCACTTCTTCGCCAAGGGTGTGTCGGACCCCTACATGGTGAGCGTGGCCAATGGAGGCACGGTACCCGACGATGTTGTCGACAGTGTTGGAGAGAAGTTACAACAGGCTTTCGGTTATTACAAAGAGAAACTTGCTGAGGACTTCGACAAAGGGTTCGATGAGCTCATGCTCGTTGACGACATGACCACCCGGGAGTACCTGAAGCGAGGCGGACCCAAGGGAGAGGCGCCCAAGTATGACTTTTTCGCCATCCAATGGATGGAGACACAAAACACCGGGACGAACCTGTTTGACCAGGCGTTTTCTGAAAGCGTCATTGACTCGTTTGACTTTGACAACCCGACAAAGCCCGAGTGGTACTGCATTGAGGGAGGAACATCACTTTTGGTGGACGCCATGGAAGAAACCCTTGTCCACAAGGTACAGAACAACAAGAGAGTTGATGCCATTTCCATTGACTTAGACGCTCCGGATGATGGGAACATGTCGGTCAGGATAGGCGGAAAGGATTACTCCGGATATAGCACCGTCTTCAACACCACCGCTCTGGGCTGTCTTGACCGCATGGATCTGCGTGGTCTCAACTTGCACCCTACTCAGGTAGATGCCATTCGATGTCTGCACTATGATAACTCGACCAAGGTGGCTCTCAAGTTTAGCTACCCGTGGTGGATCAAGGACTGTGGCATCACTTGCGGTGGCGCAGCCTCGACTGATCTGCCTCTACGAACTTGCGTTTACCCATCATACAACTTGGACGATGGCGATGGTGAGGCTGTTCTGCTTGCCTCATACACTTGGTCTCAGGACGCAACTCGCATTGGATCGTTGGTGAAGGATGCTCCACCCCAGCCGCCTAAGGAGGATGAGCTCGTCGAGCTGATCCTGCAGAATCTGGCCCGCCTGCACGCTGAGCATATGACCTACGAGAAGATTAAGGAGGCTTACACGGGCGTATATCACGCCTATTGCTGGGCTAATGATCCCAATGTCGGTGGTGCTTTCGCCCTCTTCGGTCCTGGCCAGTTCAGCAATCTGTATCCATACCTGATGCGGCCAGCGGCGGGCGGCAAGTTCCATATTGTCGGAGAGGCATCTAGTGTGCATCACGCCTGGATTATAGGTTCCTTGGAGAGCGCTTACACCGCTGTTTACCAGTTCCTGTACAAGTACAAGATGTGGGATTACTTGAGGCTGTTGTTGGAGCGCTGGCAGTATGGTCTCCAGGAGTTAGAGACGGGGAAGCACGGGACGGCTCATTTGCAGTTTATTTTGGGTTCACTTCCAAAGGAGTACCAGGTGAAGGTCTAA

**Figure S2. pGEX-4T1-LO vector map.** (A) The pGEX-4T1 bacterial vector is used to express GST-fused proteins. The vector has a multiple cloning region that contains several restriction sites. In orange: LO gene was added between BamHI and EcoRI restriction sites. Between the GST tag and the cloning sites, there is a thrombin cleaving site that allows GST removal. It also has a lac promoter for induction with IPTG and a gene that attributes resistance to ampicillin. (B) GST-rLO DNA sequence cloned into a pGEX-4T1 vector with GST highlighted in blue and rLO in green.

**Figure S3. Purification of rLO.** (A) Chromatographic profile of rLO purification by affinity chromatography. In blue: enzymatic activity of the fractions collected. In red: Absorbance of the samples at 280 nm. The firsts fractions The first fractions correspond to the crude extract. After washing the column, the enzyme activity falls and rises again when the elution of rLO begins

**Table S2. mass spectrometry analyses and blast p results**

| **Select for downloading or viewing reports** | [**Description**](https://blast.ncbi.nlm.nih.gov/Blast.cgi) | [**Scientific Name**](https://blast.ncbi.nlm.nih.gov/Blast.cgi) | [**Max Score**](https://blast.ncbi.nlm.nih.gov/Blast.cgi?CMD=Get&ADV_VIEW=yes&ADV_VIEW=on&ALIGNMENTS=100&ALIGNMENT_VIEW=Pairwise&CDD_RID=PC3ZB53P013&CDD_SEARCH_STATE=1&CONFIG_DESCR=ClustMemNbr,ClustComn,Ds,Sc,Ms,Ts,Cov,Eval,Idnt,AccLen,Acc&DATABASE_SORT=0&DESCRIPTIONS=10&DYNAMIC_FORMAT=on&FIRST_QUERY_NUM=0&FORMAT_NUM_ORG=1&FORMAT_OBJECT=Alignment&FORMAT_PAGE_TARGET=&FORMAT_TYPE=HTML&GET_SEQUENCE=yes&I_THRESH=&LINE_LENGTH=60&MASK_CHAR=2&MASK_COLOR=1&NCBI_GI=yes&NEW_VIEW=yes&NUM_OVERVIEW=10&PAGE=Proteins&QUERY_INDEX=0&QUERY_NUMBER=0&RESULTS_PAGE_TARGET=&RID=PC3ZBYJ1016&SHOW_LINKOUT=yes&SHOW_OVERVIEW=yes&STEP_NUMBER=&WORD_SIZE=5&ADV_VIEW=on&DISPLAY_SORT=1&HSP_SORT=1) | [**Total Score**](https://blast.ncbi.nlm.nih.gov/Blast.cgi?CMD=Get&ADV_VIEW=yes&ADV_VIEW=on&ALIGNMENTS=100&ALIGNMENT_VIEW=Pairwise&CDD_RID=PC3ZB53P013&CDD_SEARCH_STATE=1&CONFIG_DESCR=ClustMemNbr,ClustComn,Ds,Sc,Ms,Ts,Cov,Eval,Idnt,AccLen,Acc&DATABASE_SORT=0&DESCRIPTIONS=10&DYNAMIC_FORMAT=on&FIRST_QUERY_NUM=0&FORMAT_NUM_ORG=1&FORMAT_OBJECT=Alignment&FORMAT_PAGE_TARGET=&FORMAT_TYPE=HTML&GET_SEQUENCE=yes&I_THRESH=&LINE_LENGTH=60&MASK_CHAR=2&MASK_COLOR=1&NCBI_GI=yes&NEW_VIEW=yes&NUM_OVERVIEW=10&PAGE=Proteins&QUERY_INDEX=0&QUERY_NUMBER=0&RESULTS_PAGE_TARGET=&RID=PC3ZBYJ1016&SHOW_LINKOUT=yes&SHOW_OVERVIEW=yes&STEP_NUMBER=&WORD_SIZE=5&ADV_VIEW=on&DISPLAY_SORT=2&HSP_SORT=1) | [**Query Cover**](https://blast.ncbi.nlm.nih.gov/Blast.cgi?CMD=Get&ADV_VIEW=yes&ADV_VIEW=on&ALIGNMENTS=100&ALIGNMENT_VIEW=Pairwise&CDD_RID=PC3ZB53P013&CDD_SEARCH_STATE=1&CONFIG_DESCR=ClustMemNbr,ClustComn,Ds,Sc,Ms,Ts,Cov,Eval,Idnt,AccLen,Acc&DATABASE_SORT=0&DESCRIPTIONS=10&DYNAMIC_FORMAT=on&FIRST_QUERY_NUM=0&FORMAT_NUM_ORG=1&FORMAT_OBJECT=Alignment&FORMAT_PAGE_TARGET=&FORMAT_TYPE=HTML&GET_SEQUENCE=yes&I_THRESH=&LINE_LENGTH=60&MASK_CHAR=2&MASK_COLOR=1&NCBI_GI=yes&NEW_VIEW=yes&NUM_OVERVIEW=10&PAGE=Proteins&QUERY_INDEX=0&QUERY_NUMBER=0&RESULTS_PAGE_TARGET=&RID=PC3ZBYJ1016&SHOW_LINKOUT=yes&SHOW_OVERVIEW=yes&STEP_NUMBER=&WORD_SIZE=5&ADV_VIEW=on&DISPLAY_SORT=4&HSP_SORT=0) | [**E value**](https://blast.ncbi.nlm.nih.gov/Blast.cgi?CMD=Get&ADV_VIEW=yes&ADV_VIEW=on&ALIGNMENTS=100&ALIGNMENT_VIEW=Pairwise&CDD_RID=PC3ZB53P013&CDD_SEARCH_STATE=1&CONFIG_DESCR=ClustMemNbr,ClustComn,Ds,Sc,Ms,Ts,Cov,Eval,Idnt,AccLen,Acc&DATABASE_SORT=0&DESCRIPTIONS=10&DYNAMIC_FORMAT=on&FIRST_QUERY_NUM=0&FORMAT_NUM_ORG=1&FORMAT_OBJECT=Alignment&FORMAT_PAGE_TARGET=&FORMAT_TYPE=HTML&GET_SEQUENCE=yes&I_THRESH=&LINE_LENGTH=60&MASK_CHAR=2&MASK_COLOR=1&NCBI_GI=yes&NEW_VIEW=yes&NUM_OVERVIEW=10&PAGE=Proteins&QUERY_INDEX=0&QUERY_NUMBER=0&RESULTS_PAGE_TARGET=&RID=PC3ZBYJ1016&SHOW_LINKOUT=yes&SHOW_OVERVIEW=yes&STEP_NUMBER=&WORD_SIZE=5&ADV_VIEW=on&DISPLAY_SORT=0&HSP_SORT=0) | [**Per. Ident**](https://blast.ncbi.nlm.nih.gov/Blast.cgi?CMD=Get&ADV_VIEW=yes&ADV_VIEW=on&ALIGNMENTS=100&ALIGNMENT_VIEW=Pairwise&CDD_RID=PC3ZB53P013&CDD_SEARCH_STATE=1&CONFIG_DESCR=ClustMemNbr,ClustComn,Ds,Sc,Ms,Ts,Cov,Eval,Idnt,AccLen,Acc&DATABASE_SORT=0&DESCRIPTIONS=10&DYNAMIC_FORMAT=on&FIRST_QUERY_NUM=0&FORMAT_NUM_ORG=1&FORMAT_OBJECT=Alignment&FORMAT_PAGE_TARGET=&FORMAT_TYPE=HTML&GET_SEQUENCE=yes&I_THRESH=&LINE_LENGTH=60&MASK_CHAR=2&MASK_COLOR=1&NCBI_GI=yes&NEW_VIEW=yes&NUM_OVERVIEW=10&PAGE=Proteins&QUERY_INDEX=0&QUERY_NUMBER=0&RESULTS_PAGE_TARGET=&RID=PC3ZBYJ1016&SHOW_LINKOUT=yes&SHOW_OVERVIEW=yes&STEP_NUMBER=&WORD_SIZE=5&ADV_VIEW=on&DISPLAY_SORT=3&HSP_SORT=3) | [**Acc. Len**](https://blast.ncbi.nlm.nih.gov/Blast.cgi) | **Accession** |
| --- | --- | --- | --- | --- | --- | --- | --- | --- | --- |
| Select seq gb\|AAQ11414.1\| | [L-amino acid oxidase [Trichoderma pseudokoningii]](https://blast.ncbi.nlm.nih.gov/Blast.cgi#alnHdr_AAQ11414) | [Trichoderma pseudokoningii](https://www.ncbi.nlm.nih.gov/Taxonomy/Browser/wwwtax.cgi?id=317029) | 93.2 | 291 | 93% | 4e-17 | 51.33% | 447 | [AAQ11414.1](https://www.ncbi.nlm.nih.gov/protein/AAQ11414.1?report=genbank&log$=prottop&blast_rank=1&RID=PC3ZBYJ1016) |
| Select seq emb\|CAC81784.1\| | [putative L-aminoacid oxidase [Trichoderma lixii]](https://blast.ncbi.nlm.nih.gov/Blast.cgi#alnHdr_CAC81784) | [Trichoderma lixii](https://www.ncbi.nlm.nih.gov/Taxonomy/Browser/wwwtax.cgi?id=1491472) | 93.2 | 297 | 97% | 4e-17 | 51.33% | 447 | [CAC81784.1](https://www.ncbi.nlm.nih.gov/protein/CAC81784.1?report=genbank&log$=prottop&blast_rank=2&RID=PC3ZBYJ1016) |
| Select seq ref\|XP_056025961.1\| | [flavin containing amine oxidoreductase domain-containing protein [Trichoderma breve]](https://blast.ncbi.nlm.nih.gov/Blast.cgi#alnHdr_XP_056025961) | [Trichoderma breve](https://www.ncbi.nlm.nih.gov/Taxonomy/Browser/wwwtax.cgi?id=2034170) | 93.2 | 295 | 97% | 6e-17 | 51.33% | 617 | [XP_056025961.1](https://www.ncbi.nlm.nih.gov/protein/XP_056025961.1?report=genbank&log$=prottop&blast_rank=3&RID=PC3ZBYJ1016) |
| Select seq gb\|OPB45577.1\| | [L-amino acid oxidase [Trichoderma guizhouense]](https://blast.ncbi.nlm.nih.gov/Blast.cgi#alnHdr_OPB45577) | [Trichoderma guizhouense](https://www.ncbi.nlm.nih.gov/Taxonomy/Browser/wwwtax.cgi?id=1491466) | 93.2 | 297 | 97% | 6e-17 | 51.33% | 617 | [OPB45577.1](https://www.ncbi.nlm.nih.gov/protein/OPB45577.1?report=genbank&log$=prottop&blast_rank=4&RID=PC3ZBYJ1016) |
| Select seq gb\|KKP06428.1\| | [hypothetical protein THAR02_01479 [Trichoderma harzianum]](https://blast.ncbi.nlm.nih.gov/Blast.cgi#alnHdr_KKP06428) | [Trichoderma harzianum](https://www.ncbi.nlm.nih.gov/Taxonomy/Browser/wwwtax.cgi?id=5544) | 93.2 | 297 | 97% | 6e-17 | 51.33% | 618 | [KKP06428.1](https://www.ncbi.nlm.nih.gov/protein/KKP06428.1?report=genbank&log$=prottop&blast_rank=5&RID=PC3ZBYJ1016) |
| Select seq gb\|KAK4069404.1\| | [hypothetical protein Trihar35433_5983 [Trichoderma harzianum]](https://blast.ncbi.nlm.nih.gov/Blast.cgi#alnHdr_KAK4069404) | [Trichoderma harzianum](https://www.ncbi.nlm.nih.gov/Taxonomy/Browser/wwwtax.cgi?id=5544) | 93.2 | 295 | 97% | 6e-17 | 51.33% | 617 | [KAK4069404.1](https://www.ncbi.nlm.nih.gov/protein/KAK4069404.1?report=genbank&log$=prottop&blast_rank=6&RID=PC3ZBYJ1016) |
| Select seq ref\|XP_024771776.1\| | [hypothetical protein M431DRAFT_510303 [Trichoderma harzianum CBS 226.95]](https://blast.ncbi.nlm.nih.gov/Blast.cgi#alnHdr_XP_024771776) | [Trichoderma harzianum CBS 226.95](https://www.ncbi.nlm.nih.gov/Taxonomy/Browser/wwwtax.cgi?id=983964) | 93.2 | 302 | 97% | 7e-17 | 51.33% | 618 | [XP_024771776.1](https://www.ncbi.nlm.nih.gov/protein/XP_024771776.1?report=genbank&log$=prottop&blast_rank=7&RID=PC3ZBYJ1016) |
| Select seq gb\|QYT04843.1\| | [L-Lysine alpha-oxidase [Trichoderma simmonsii]](https://blast.ncbi.nlm.nih.gov/Blast.cgi#alnHdr_QYT04843) | [Trichoderma simmonsii](https://www.ncbi.nlm.nih.gov/Taxonomy/Browser/wwwtax.cgi?id=1491479) | 93.2 | 300 | 97% | 7e-17 | 51.33% | 617 | [QYT04843.1](https://www.ncbi.nlm.nih.gov/protein/QYT04843.1?report=genbank&log$=prottop&blast_rank=8&RID=PC3ZBYJ1016) |
| Select seq gb\|KAF3057694.1\| | [putative bifunctional amine oxidase [Trichoderma lentiforme]](https://blast.ncbi.nlm.nih.gov/Blast.cgi#alnHdr_KAF3057694) | [Trichoderma lentiforme](https://www.ncbi.nlm.nih.gov/Taxonomy/Browser/wwwtax.cgi?id=1567552) | 92.8 | 293 | 97% | 7e-17 | 51.33% | 618 | [KAF3057694.1](https://www.ncbi.nlm.nih.gov/protein/KAF3057694.1?report=genbank&log$=prottop&blast_rank=9&RID=PC3ZBYJ1016) |
| Select seq pdb\|7C3I\|A | [Chain A, L-lysine oxidase [Trichoderma viride]](https://blast.ncbi.nlm.nih.gov/Blast.cgi#alnHdr_7C3I_A) | [Trichoderma viride](https://www.ncbi.nlm.nih.gov/Taxonomy/Browser/wwwtax.cgi?id=5547) | 90.9 | 222 | 82% | 3e-16 | 50.44% | 540 | [7C3I_A](https://www.ncbi.nlm.nih.gov/protein/7C3I_A?report=genbank&log$=prottop&blast_rank=10&RID=PC3ZBYJ1016) |

**Table S3. Substrate specificity data of rLO for different L-amino acids.**

| **Amino acid*** | **Activity (U/mL)** | **Relative Activity (%)** |
| --- | --- | --- |
| **L-lysine** | 5.237 | 100 |
| **L-arginine** | 1.591 | 30.4 |
| **L-phenylalanine** | 1.162 | 22.2 |

*rLO was ineffective with other L-amino acids

**Figure S4. Enzymatic characterization of rLO.** (A) Effect of pH on rLO in percentage of relative activity. The tested pHs were: 2, 3, 4, 5, 6, 7, 7.5, 8, 8.6, 9, and 10 with different buffers. (B) Effect of temperature on rLO activity. The incubation temperatures tested were 4, 10, 20, 25, 37, 50, 60, 70, 80, and 100 ^o^C. (C) Effect of substrate concentration on rLO activity. The concentrations used were: 0.1, 0.5, 1, 5, 10, 50, 100, 200, and 300 mM of L-lysine. (D) Graph of substrate concentration versus initial velocity (V0) to determine the Michaelis-Menten constant (K_M_/ K_0.5_) and Maximum velocity (V_máx_).

**Table S4.** Kinetic parameters were calculated for rLO with L-lysine as substrate.

| **Parameter** | **rLO** |
| --- | --- |
| **K_M_ (mM)** | 28.84 |
| **Vmáx (µmol/min)** | 0.01569 |
| **n_H_** | 0.933 |

**Figure S5. Comparison of the effect in the viability of rLO****, rLO^GST-^ and GST in Jurkat cells.**

GST-tagged rLO (rLO) was produced in *E. coli* with pGEX-4T1-LO vector and the untagged protein (rLO^GST-^) was obtained using a thrombin cleavage site between GST and rLO. GST was produced in *E. coli* transformed with pGEX-4T1 empty vector. ATO was used as a positive control. (A, D) Effect of treatments on cell viability. (B, E) Percentage of positive cells for Annexin V only (cells in apoptosis). (C, F) Percentage of positive cells in late apoptosis (double marked for Annexin V and PI). All treatments were carried out with enzymes at a concentration of 1 mU/mL

**Table S5.** List of signaling pathways, primers, and genes evaluated by qPCR

| **Pathway** | **Gene** | **Sense** | **Sequence** |
| --- | --- | --- | --- |
| Autophagy | MAP1LC3A | F | AACATGAGCGAGTTGGTCAAG |
| Autophagy | MAP1LC3A | R | GCTCGTAGATGTCCGCGAT |
| JNK | TP53 | F | CCTCAGCATCTTATCCGAGTGG |
| JNK | TP53 | R | TGGATGGTGGTACAGTCAGAGC |
| Autophagy | ATG7 | F | CGTTGCCCACAGCATCATCTTC |
| Autophagy | ATG7 | R | CACTGAGGTTCACCATCCTTGG |
| JNK | Cyclin E | F | TGTGTCCTGGATGTTGACTGCC |
| JNK | Cyclin E | R | CTCTATGTCGCACCACTGATACC |
| JNK | Rbp | F | CTCTCGTCAGGCTTGAGTTTG |
| JNK | Rbp | R | GACATCTCATCTAGGTCAACTGC |
| p38 | p21 | F | AGGTGGACCTGGAGACTCTCAG |
| p38 | p21 | R | TCCTCTTGGAGAAGATCAGCCG |
| p38 | Elk1 | F | GCTGCCTCCTAGCATTCACTTC |
| p38 | Elk1 | R | CCACGCTGATAGAAGGGATGTG |
| p38 | ATF2 | F | GGTAGCGGATTGGTTAGGACTC |
| p38 | ATF2 | R | TGCTCTTCTCCGACGACCACTT |
| p38 | cJUN | F | CCTTGAAAGCTCAGAACTCGGAG |
| p38 | cJUN | R | TGCTGCGTTAGCATGAGTTGGC |
